# Associations Between Common and Rare Exonic Genetic Variants and Serum Levels of 20 Cardiovascular-Related Proteins: The Tromsø Study

**DOI:** 10.1161/CIRCGENETICS.115.001327

**Published:** 2016-08-16

**Authors:** Terry Solomon, Erin N. Smith, Hiroko Matsui, Sigrid K. Braekkan, Tom Wilsgaard, Inger Njølstad, Ellisiv B. Mathiesen, John-Bjarne Hansen, Kelly A. Frazer, INVENT Consortium

**Affiliations:** From the Biomedical Sciences Graduate Program, University of California, San Diego, La Jolla (T.S.), Department of Pediatrics, Rady’s Children’s Hospital, San Diego, La Jolla, CA (E.N.S., H.M., K.A.F.); Institute for Genomic Medicine, University of California, San Diego, La Jolla (K.A.F.); Department of Clinical Medicine, K.G. Jebsen Thrombosis Research and Expertise Centre (TREC) (E.N.S., S.K.B., I.N., E.B.M., J.-B.H., K.A.F.), Department of Community Medicine (T.W., I.N.), and Brain and Circulation Research Group, Department of Clinical Medicine (E.B.M.), UiT The Arctic University of Norway; and Division of Internal Medicine, University Hospital of North Norway, Tromsø (S.K.B., J.-B.H.).

**Keywords:** biomarker, coronary artery disease, exome, human, protein, venous thromboembolism

## Abstract

**Background—:**

Genetic variation can be used to study causal relationships between biomarkers and diseases. Here, we identify new common and rare genetic variants associated with cardiovascular-related protein levels (protein quantitative trait loci [pQTLs]). We functionally annotate these pQTLs, predict and experimentally confirm a novel molecular interaction, and determine which pQTLs are associated with diseases and physiological phenotypes.

**Methods and Results—:**

As part of a larger case–control study of venous thromboembolism, serum levels of 51 proteins implicated in cardiovascular diseases were measured in 330 individuals from the Tromsø Study. Exonic genetic variation near each protein’s respective gene (*cis*) was identified using sequencing and arrays. Using single site and gene-based tests, we identified 27 genetic associations between pQTLs and the serum levels of 20 proteins: 14 associated with common variation in *cis*, of which 6 are novel (ie, not previously reported); 7 associations with rare variants in *cis*, of which 4 are novel; and 6 associations in *trans*. Of the 20 proteins, 15 were associated with single sites and 7 with rare variants. *cis*-pQTLs for kallikrein and F12 also show *trans* associations for proteins (uPAR, kininogen) known to be cleaved by kallikrein and with NTproBNP. We experimentally demonstrate that kallikrein can cleave proBNP (NTproBNP precursor) in vitro. Nine of the pQTLs have previously identified associations with 17 disease and physiological phenotypes.

**Conclusions—:**

We have identified *cis* and *trans* genetic variation associated with the serum levels of 20 proteins and utilized these pQTLs to study molecular mechanisms underlying disease and physiological phenotypes.

## Abstract 2

Supplemental Digital Content is available in the text.

Recent advances in genetics have yielded an unprecedented number of loci associated with disease and are beginning to yield mechanistic insight, such as with the IRX3/5 association with body mass index, which revealed brown adipose as an important regulator of body weight.^1^ Genetic variation underlying molecular phenotypes, such as proteins and transcript expression levels, can be important tools in constructing the effects of genetic variations into pathways, ultimately resulting in physiological understanding of diseases.^2^ Protein levels, in particular, may be more informative for understanding disease because there is often a poor correlation between transcript and protein levels.^3^ Several previous studies^4–6^ have systematically identified genetic variations associated with protein levels and isoforms (protein quantitative trait loci [pQTLs]). Although most studies have focused on common variation (minor allele frequency ≥5%), rare variants, which can show strong loss of function effects, can be useful in understanding causality and pinpointing drug targets, such as deletion mutations in *PSCK9* that abolish the PSCK9 protein and reduce low-density lipoprotein cholesterol levels.^7^ Systematic screening for rare variation influencing a wide variety of proteins, however, has not yet been performed.

**Editorial, see p 318**

**Clinical Perspective on p 383**

Genetic variation is also useful in identifying causal relationship between biomarkers and diseases using tools such as Mendelian randomization^8^ and could be used to ascertain how risk factors differentially affect various diseases, as well as trace causal pathways between risk loci and disease. We are investigating risk factors for cardiovascular diseases, including myocardial infarction (MI) and venous thromboembolism (VTE) in the Tromsø Study,^9^ a longitudinal prospective cohort study. We previously assayed 51 cardiovascular-related proteins in 419 first-ever MI cases and 398 controls in serum collected years before the MI event.^10^ Of the proteins measured, 17 were predictors for MI when considered individually after adjusting for traditional risk factors. Genetic variation associated with these protein levels could be used to study underlying mechanisms of cardiovascular diseases.

Here, using whole exome sequencing data and HumanCoreExome BeadChips, we investigate whether genetic variants are associated with the serum levels of the same 51 cardiovascular-related proteins in 330 individuals chosen from the Tromsø Study because they did or did not go on to develop VTE during the 18 years of follow-up (mean time to VTE of 9 years). The serum samples were collected at study entry enabling us to identify pQTLs associated with baseline protein levels. We perform both common and rare variation association analyses to identify *cis*-pQTLs. Further characterization of the *cis*-pQTLs to determine whether they also act as *trans*-pQTLs with any of the other 51 cardiovascular-related proteins, recapitulated well-established physiological relationships between F12, kallikrein, uPAR, kininogen, and a recent genetic association with NTproBNP. We experimentally confirmed an inferred physiological interaction from the *trans*-pQTLs by showing that kallikrein cleaves proBNP in vitro. We then examine genetic associations from genome-wide association studies on coronary artery disease (CAD) and VTE, as well as published literature to identify physiological and disease associations.

## Methods

### The Tromsø Study

The Tromsø Study is a prospective, single-site, cohort study of the inhabitants of Tromsø, Norway. In 1994 to 1995, 27 158 individuals filled out epidemiological surveys and donated (nonfasting) blood to the National CONOR Biobank.^9^ These individuals were followed until 2013, with repeated surveys and identified in national registries that report various diseases and causes of death. In 2013, we identified individuals who, between 1995 and 2013, had had an incident of VTE or death due to VTE, regardless of other comorbidities. We chose age- and sex-matched controls randomly from the cohort. These samples were chosen for a currently ongoing case–control study of VTE. DNA and protein levels were ascertained from the blood collected in 1994.

For this specific study, blood and nonfasting serum samples were collected from 330 healthy individuals (166 males and 164 females) aged 45 to 75 years (Table I in the Data Supplement). There were 196 individuals diagnosed with VTE between the study entry (1994–1995) and the 18-year follow-up period (2013) and 134 controls without development of VTE during this period. Aspirin usage and other medication information were not collected for the Tromsø study. DNA was isolated from the blood for genotyping, and serum samples were used to assay protein levels. The regional committee for medical and health research ethics in North Norway approved the study, and all participants gave informed written consent.

### Protein Quantification

Protein levels were quantified using the same methods and at the same time as our previous MI study,^10^ but the samples from people who went on to develop VTE were not included in that study. Briefly, the literature was searched to create a list of >900 cardiovascular-related proteins that might be potential biomarkers for MI and atherosclerosis. This list was then prioritized to 165 candidate proteins, of which 51 had sufficient commercially available reagents (2 antibodies and purified protein for control) in order for Tethys Bioscience, Inc (Emeryville, CA) to perform successful sandwich enzyme-linked immunosorbent assays (Methods section and Table II in the Data Supplement). All protein levels were quantile normalized and mapped to the normal distribution using qnorm in R, and significance was tested using *Z* scores.

### Variant Identification and Annotation

Genotypes were determined using exome sequencing (n=243) or exome genotyping arrays (n=87). Sequences were mapped and called using the Burrows-Wheeler Aligner^11^ and Genome Analysis Toolkit,^12^ imputed to the 1000 Genomes Project^13^ using Beagle,^14^ and functionally annotated for predicted effect and regulatory regions (Methods section in the Data Supplement).

### Statistical Analysis

Associations were performed using the Efficient and Parallelizable Association Container Toolbox.^15^ We used sex, age at study entry, body mass index at study entry, genotyping platform, and VTE case–control status as covariates. Three covariates (age at serum collection, sex and body mass index at serum collection) were associated, respectively, with 10, 10, and 13 of the phenotypes (the 51 protein serum levels) when performing linear regressions, defined as having a false-discovery rate-adjusted *P* value of <0.05, and they were included for consistency.

**Figure 1.**
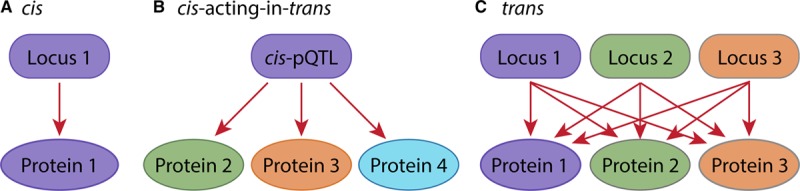
Overview of the 3 stages of association analyses. **A**, *cis*: for each of the 51 phenotypes (protein levels), we tested the variants located in the *cis* gene loci for associations with their respective protein level, (**B**) *cis*-acting-in-*trans*: we tested the significant *cis*-protein quantitative trait loci (pQTLs) from stage 1 for *trans* effects against each of the 50 other protein levels, and (**C**) *trans*: we tested all variants in the 50 *cis* loci (C3 and C3b share the same locus) for association with each of the 51 protein levels.

For common variants (MAF≥1%), we used the Efficient Mixed Model Association eXpedited^16^ (a mixed model implemented in the Efficient and Parallelizable Association Container Toolbox^15^), using q.emmax to test for single-site association. For *cis* associations, we included any imputed common variants located within the interval surrounding and including the gene (±500 kb from transcript start and stop positions) that encodes the protein(s) being tested (C3 and C3b share the same locus). For *cis*-acting-in-*trans* associations, we tested all significantly associated common *cis* variants against each of the other 50 phenotypes. For *trans* associations, we tested the 100 378 common variants found in the 50 intervals against each of the 51 phenotypes (Figure 1).

The optimal Sequence Kernel Association Test^17^ was used to test clusters of rare variants (MAF≤5%) for association as implemented in the Efficient and Parallelizable Association Container Toolbox, using the skat-o version of the mmskat test. Rare variants were classified in 3 ways: (1) MAF≤5%, all rare variants located within the gene body and 2kb upstream; (2) deleterious, all rare variants located in the gene body and the 2-kb upstream region that were annotated as stop-gain, stop-loss, start-loss, essential splice site disruption, frame-shift causing, or nonsynonymous using Variant Effect Predictor annotations; and (3) Combined Annotation Dependent Depletion (CADD)-score, all rare variants in the gene or the 2-kb upstream region with a Phred-scaled c-score of >10, as determined by Kircher et al.^18^

We corrected for multiple testing by permuting the phenotype–genotype relationship 1000× and for each permutation performing all variant-phenotype tests for each association type separately (eg, *cis*, *cis*-acting-in-*trans*, or *trans*).^19^ We obtained the lowest *P* value from each permutation across all phenotypes and created a null distribution of minimum *P* values. An association was considered significant (family-wise *P*<0.05) if the nominal *P* value was <95% of the null distribution (Table III in the Data Supplement).

To test for multiple, independent variants in the same locus, the top variant was included as a covariate until there was no longer a significant association (family-wise *P*<0.05) detected for that protein.

### Power Calculations

We calculated power using an equation from the Abecasis laboratory (http://genome.sph.umich.edu/wiki/Power_Calculations:_Quantitative_Traits) for common variants and the Sequence Kernel Association Test R package^20^ for rare variants. We had 80% power to detect effects (*R*^2^) down to 0.113 for the *cis*, common-variant analysis and effects (β) of 1.25 for the *cis*, rare-variant analysis (assuming that 50% of the variants are causal), which is comparable to other pQTL studies.^4–6,21,22^ Further details are in the Data Supplement (Methods section, Figures I and II, and Table IV in the Data Supplement).

### Clinical and Molecular Phenotype Association

Significant pQTLs from this study were queried against the expression Quantitative Trait Loci (eQTLs) found by Schadt et al^23^ in liver cells and the Gene-Tissue Expression database^24^ (version 4, build 200, accessed at http://www.gtexportal.org/home/) for all tissue types. In addition, we determined whether they (or a variant in linkage disequilibrium [LD]) overlapped any of the variants identified as pQTLs in 5 similar-sized independent studies that investigated protein levels in serum^4^ or plasma.^4–6,22,25^ We examined pQTLs for clinical significance by determining whether the variant has been previously identified and submitted to the Online Mendelian Inheritance in Man,^26^ the genome-wide association study (GWAS) Catalog,^27^ or the Genome-Wide Repository of Associations between Single nucleotide polymorphisms and Phenotypes v2.0.^28^ We identified pQTLs that were also significant in large meta-analyses of individuals of European descent for CAD or VTE. Data on CAD was downloaded from http://www.cardiogramplusc4d.org. For this analysis, we only used the results from the Coronary Artery Disease Genome wide Replication and Meta-analysis (CARDIoGRAM) genome-wide association study^29^ because these individuals are of European descent. Data on VTE was shared by the International Network against Thrombosis (INVENT Consortium).^30^

### In Vitro Assay of proBNP Cleavage

We obtained native kallikrein from human plasma from EMD-Millipore (Darmstadt, Germany; cat no. 420307); recombinant proBNP from Abcam (Cambridge, Ma; cat no. ab151881); and the kallikrein inhibitor, H-D-Phe-Phe-Arg-chloromethyl ketone (PPACK II), from Santa Cruz Biotechnology (Dallas, TX; cat no. sc-203215). A total of 354 ng (374 nmol/L) of kallikrein was incubated with 80 ng (606 nmol/L) of proBNP with and without 26.5 ng (36.4 μmol/L) of PPACK II for 30, 60, and 90 minutes at 37°C. The reactions were stopped by adding 4× lithium dodecyl sulfate sample buffer and dithiothreitol, and heating them for 2 minutes at 85°C. The proteins were run on a Tricine-SDS–PAGE gel from ThermoFisher (Waltham, MA), and either detected using the SilverQuest Silver Staining Kit from ThermoFisher or transferred to a polyvinylidene difluoride membrane and detected using an anti-BNP antibody from Novus Biologicals (Littleton, Co; cat no. NB100-62133) and chemiluminescence.

## Results

### Study Overview

The subjects were chosen as a substudy from an ongoing case–control study examining the genetics of VTE, and includes 196 individuals who developed VTE during the 18-year follow-up and 134 individuals who did not (Table I in the Data Supplement). Serum was assayed for the levels of 51 proteins using enzyme-linked immunosorbent assays (Table II in the Data Supplement). On average, we obtained high-quality protein measurements for 311 individuals per phenotype. We investigated whether any of the protein levels were associated with VTE case–control status and found no significant associations. Knowing that the protein levels were not statistically associated with VTE enabled us to combine the VTE cases and controls to explore the effects of genetic variation on baseline protein levels.

We performed high coverage (≈100×) exome sequencing on DNA from blood samples for 243 individuals and assayed an additional 87 with HumanCoreExome Beadchips. We identified 158 137 variants (direct genotyping and imputation) in the 50 intervals that encode the 51 proteins (Table V in the Data Supplement). The majority of imputed variants were intergenic or intronic because these variants were not already captured by the genotyping array or were outside of the exome-sequencing target regions (Table VI in the Data Supplement). There was an average of 1122 variants per locus with the *AGER* locus having the most variants (3523) and the *CD40LG* locus having the fewest (441; Table II in the Data Supplement).

### Identifying *cis*-pQTLs From Common Variants

**Figure 2.**
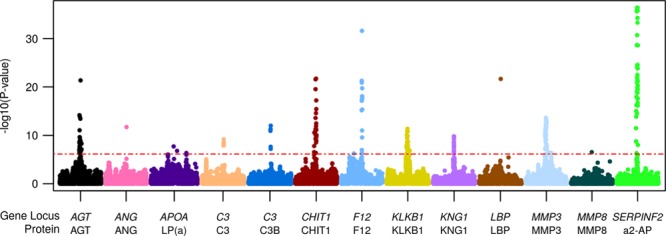
Association of *cis* variants with protein levels. Modified Manhattan plot showing the –log10 *P* values for association between variants in each *cis* locus (interval encoding protein ±500 kb) and the respective protein levels. The red dashed line indicates the study-wide significant *P*-value cutoff when only examining *cis* regions (6.9×10^−7^) for a family-wise error rate <0.05.

To identify genetic variation associated with serum protein levels, we tested for association between variants within the gene’s *cis* locus and the normalized protein level for each of the 51 protein levels, adjusting for sample relatedness and population structure using a kinship matrix and including age, sex, body mass index, genotype platform, and subsequent VTE status as covariates. Because of the high likelihood of linkage disequilibrium at the *cis* loci and slight correlations among protein levels, we accounted for multiple testing by performing permutations to obtain a family-wise error rate. We identified significant associations (adjusted *P*<0.05, nominal *P*<6.97×10^−7^; Table 1; Figure 2) for 13 of the 51 phenotypes. To test for multiple, independent associations, we performed sequential conditioning on the most highly associated variant and found 2 independent *cis* associations for LP(a). Of the 14 *cis*-pQTLs that we report, we have replicated 8 known pQTLs and identified 6 novel pQTLs. The same variant or a variant in LD (r^2^>0.5 in EUR) has been previously reported for 8 proteins with the same direction of effect that we found: AGT,^22^ C3,^5^ C3b,^5^ CHIT1,^6^ F12,^6,25^ LBP,^6^ one of the variants for LP(a),^31^ and MMP3^32^ (Table VII in the Data Supplement). Of the 6 novel pQTLs that we identified, 4 proteins have not previously been reported to have a *cis*-pQTL (a2-AP, ANG, KLKB1, and MMP8) and 2 proteins have been previously associated with a pQTL, but the variant identified here is not in LD with the previous variant (KNG1^25,6^ and LP(a)^31^). rs3373402 in *KLKB1* was previously reported to affect KLKB1 binding with kininogen (KNG1) but did not affect KLKB1 levels in plasma^33^; therefore, although this variant has been previously functionally characterized, this is a novel pQTL. We annotated the 14 pQTLs for functional effects and identified their chromatin state in the tissue where their target gene is most highly expressed (Table VIII Data Supplement). Ten of the 13 proteins are predominantly secreted by the liver. Five of the top variants are missense variants, 3 are in the untranslated regions, and 5 lie in predicted regulatory regions based on chromatin state annotations. These analyses suggest possible mechanisms of action for some of the *cis*-pQTLs.

**Table 1.**
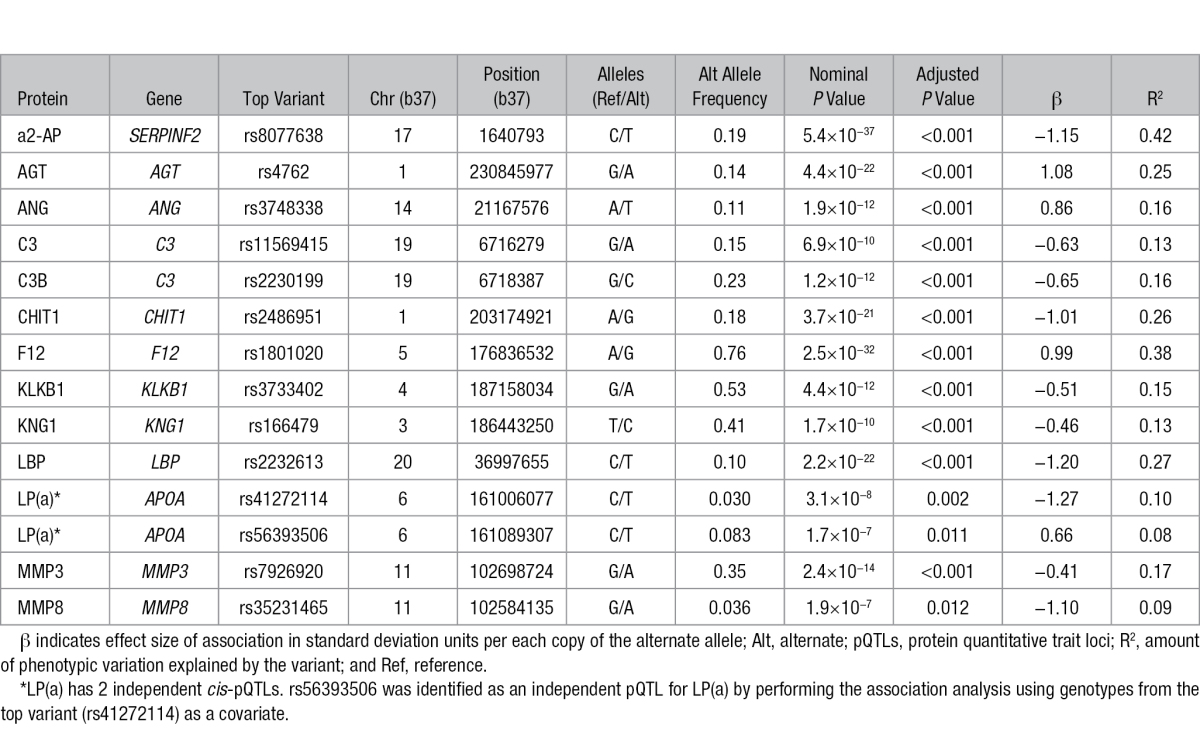
Significant cis-pQTLs From the Common-Variant Association Analysis

### Identifying *cis*-pQTLs From Rare Variation

We next tested whether the combination of multiple rare variants at each *cis*-locus was associated with protein levels. There were 3675 rare variants identified across all 50 loci. For rare variation association analyses, rare variants are grouped according to frequency or function and then jointly tested for association. Because functional prediction methods vary and it is currently unknown what method is superior,^34^ we used 3 different classifications (MAF, Deleterious, and CADD-score—see Methods section of this article). Across all loci, there was a range of 1 to 90 variants used for each method, with the MAF method having the most rare variants and CADD scores having the fewest. To account for multiple testing, we tested all the 3 classifications in each round of permutations to determine the family-wise error rate *P*-value cutoff. We performed an optimal Sequence Kernel Association Test using the same covariates as for the common-variant association. We identified 8 *cis*-pQTLs that were significant using ≥1 classifications (adjusted *P*<0.05, nominal *P*<3.72×10^−4^; Table 2; Table IX in the Data Supplement). Of these, *cis* rare variation has been associated with AGER,^35^ Fetuin A,^36^ and LP(a) levels^31^; to our knowledge, the other 5 associations are novel.

**Table 2.**
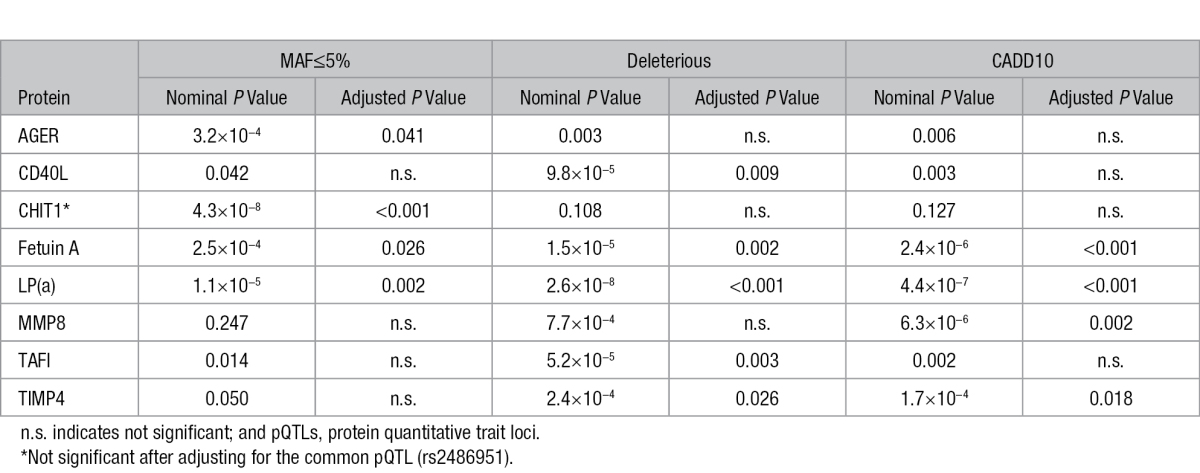
Rare Variant cis-pQTLs That Are Significant Using At Least 1 of the 3 Grouping Methods

Of the 8 proteins associated with rare variation, 3 were also associated with a common pQTL (CHIT1, LP(a), and MMP8). For LP(a) and MMP8, a common pQTL (with an MAF<5%) was also present on the list of rare variants and removal of these from the rare-variant analysis made the rare association nonsignificant (CADD nominal *P* value, 0.148 and 0.469, respectively). For CHIT1, the common pQTL had an MAF of 18% and although not on the list of rare variants, when we included this variant as a covariate in the rare-variant analysis the association was nullified (nominal *P* value=0.147). These results suggest that the rare variants in the *CHIT1* locus were associated with CHIT1 serum levels because of linkage disequilibrium with the common pQTL. Because the driving variant was common, we do not consider the CHIT1 association to be valid, resulting in 7 proteins associated with rare variants.

### Identifying *trans*-pQTLs

**Figure 3.**
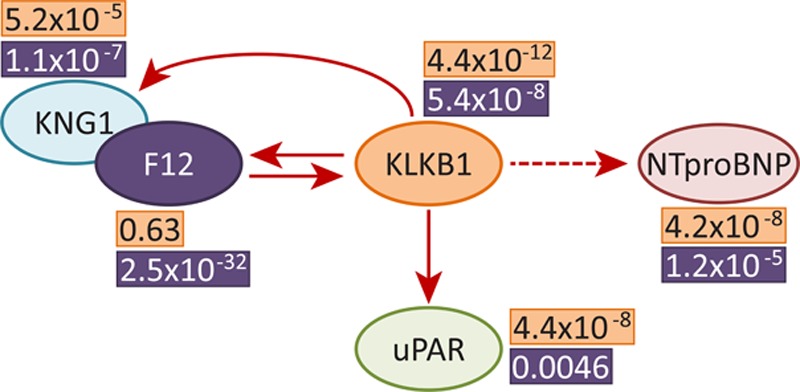
Schematic diagram showing proteins with identified *trans* associations and their nominal associations with variants in *F12* and *KLKB1*. Previously known (solid) and proposed in this study (dashed) cleavage reactions are represented with arrows. Nominal *P* values for the associations between protein levels and rs3733402 in the *KLKB1* locus and rs1801020 in the *F12* locus are shown, respectively, in orange and purple boxes next to the protein of interest.

To characterize potential downstream effects of *cis*-pQTLs, we investigated whether any of the common *cis*-pQTLs might also have *trans* effects (*cis*-acting-in-*trans*) on any of the other 50 protein levels. After permutation to obtain adjusted *P* values, we identified 2 *cis*-acting-in-*trans* loci, each of which was significantly associated with 3 proteins (adjusted *P*<0.05, nominal *P*<7.29×10^−5^; Table 3). There was significant overlap in the proteins associated with the 2 loci, and the associations were consistent with known physiological relationships between F12, KLKB1, KNG1, and uPAR, and the recently reported genetic relationship with NTproBNP^37^ (Figure 3), despite none of the protein levels being strongly correlated (Figure 3; Table X in the Data Supplement). We did not observe an association between the *cis*-pQTL for *KLKB1* and F12 protein levels, despite the known physiological relationships of KLKB1 and F12 (Figure 3). Importantly, the genetic associations of *KLKB1* and *F12* with NTproBNP suggest that KLKB1 may physiologically cleave proBNP (the NTproBNP precursor). These findings illustrate how genetic variation can be used to identify potentially novel physiological relationships among proteins.

**Table 3.**
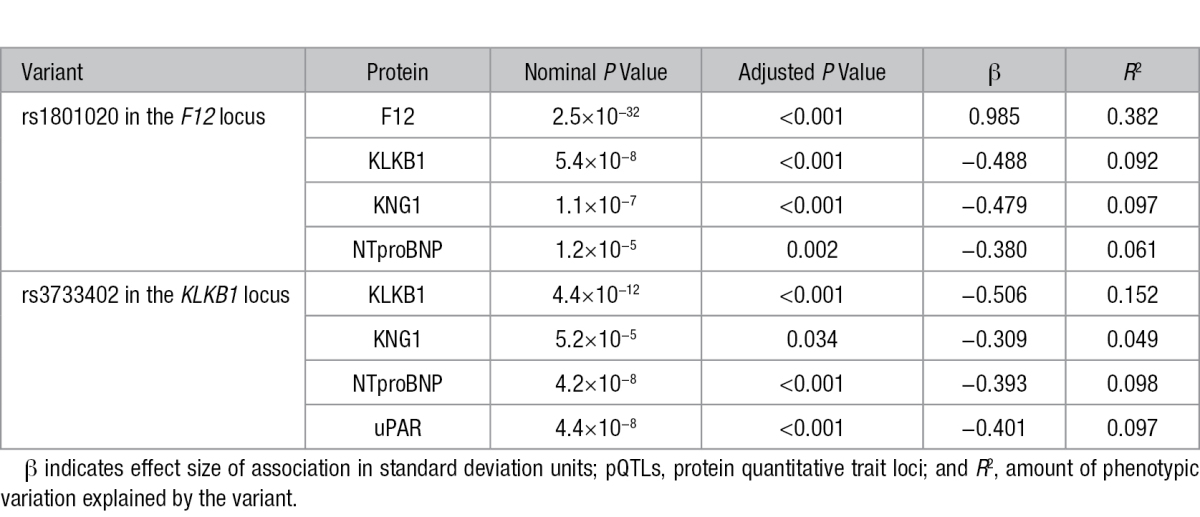
cis-pQTLs That Also Act As trans-pQTLs

We further performed a full pairwise association (*trans*) between any of the variants located in the 50 regions encoding the proteins used in this study and all 51 protein levels. After permutation adjusting (adjusted *P*<0.05, nominal *P*<1.25×10^−8^), we did not find any additional *trans* associations and none of the *cis*-acting-in-*trans* associations remained significant; however, 11 of the 14 *cis* associations remained significant.

Using a similar approach to the common variants, we tested if any of the rare variant *cis*-pQTLs were associated with any of the other 50 protein levels and did not observe any significant associations (adjusted *P*<0.05, nominal *P*<5.30×10^−5^). In addition, we tested all 50 *cis* regions against all 51 protein levels in a pairwise manner, but did not identify additional associations (adjusted *P*<0.05, nominal *P*<9.21×10^−6^), although 4 of the 8 rare *cis* associations were still significant at the more stringent threshold.

### Role of Kallikrein in proBNP Maturation

**Figure 4.**
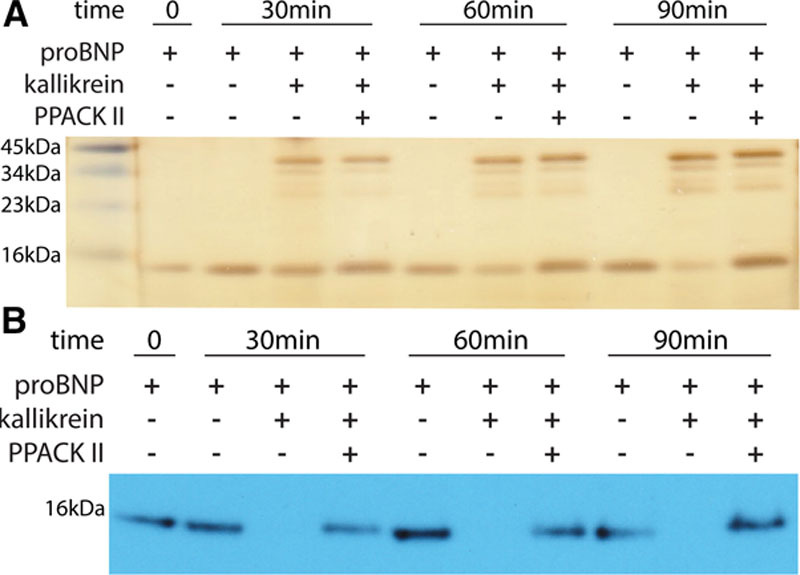
Kallikrein cleaves proBNP in vitro. **A**, A silver stain of recombinant proBNP and kallikrein incubated together for 30, 60, and 90 min with and without a kallikrein-specific inhibitor (PPACK II) and (**B**) a western blot of an identical experimental setup using an anti-BNP antibody. The silver stain binds all protein present and is a more sensitive procedure than using the anti-BNP antibody for the Western blot. We think that this explains why the amount of proBNP in the +/+/− wells visually seems to be different between the silver stain and Western blot.

We experimentally tested the *cis*-acting-in-*trans* associations suggesting that kallikrein (KLKB1) may physiologically cleave proBNP. ProBNP is produced as a propeptide that may be cleaved intracellularly into BNP and NTproBNP, 2 biomarkers for heart failure,^38^ before being secreted by cardiomyocytes in response to cardiac stress. Intracellularly, it is thought that furin or corin cleave proBNP,^39^ but it is unclear which enzyme cleaves proBNP extracellularly when it is secreted intact.^40^ To test whether kallikrein can cleave proBNP in vitro, we incubated increasing concentrations of kallikrein (74.8, 374, 748, and 1497 nmol/L) with proBNP for 1 hour at body temperature (37°C) and saw progressive depletion of proBNP levels (Figure IV in the Data Supplement). This depletion was prevented with the addition of PPACK II, a kallikrein-specific inhibitor. From this, we chose to incubate 374 nmol/L of kallikrein with proBNP for 30, 60, or 90 minutes and again, we saw that the levels of proBNP decreased (Figure 4). These results suggest that kallikrein has the ability to cleave proBNP in vivo.

### Annotation of pQTLs Using Existing Databases and GWAS

We investigated whether the 14 common pQTLs that we identified were reported as eQTLs in the Gene-Tissue Expression database (ref 24) or identified in the liver eQTL dataset from Schadt et al._23_ In the Gene-Tissue Expression database, the AGT pQTL was identified as an eQTL in 10 tissues (*P* values from of 2.0×10^−6^ to 1.3×10^−33^), the CHIT1 pQTL is an eQTL in whole blood (*P* value 4.2×10^−8^), the F12 pQTL is an eQTL in liver (*P* value 2.3×10^−10^), and the pQTL in the *SERPINF2* locus (a2-AP protein) is an eQTL in 6 tissues (*P* values from 5.3×10^−7^ to 8.8×10^−18^). In addition, the pQTLs for a2-AP, AGT, CHIT1, F12, KLKB1, and MMP3 were also identified as eQTLs for other nearby genes. In the Schadt et al’s data set rs3748338 in the *ANG* locus is in LD (r^2^=0.24) with an eQTL for ANG (rs8008440). Thus, of the 14 common pQTLs, 2 have previously been identified as an eQTL for the *cis* gene, 3 as an eQTL for both the *cis* gene and other nearby genes, and 3 as an eQTL for nearby gene(s).

We also looked up whether there are any known disease associations with the 14 pQTLs that we identified using the GWAS catalog.^27^ the Genome-Wide Repository of Associations between Single nucleotide polymorphisms and Phenotypes,^28^ and the Online Mendelian Inheritance in Man^26^ (Table VII in the Data Supplement). The 8 known pQTLs along with the kallikrein pQTL are associated with a variety of phenotypes, including age-related macular degeneration (C3b), activated partial thromboplastin times (F12), serum metabolites (KLKB1), binding of LBP to lipopolysaccharide (LBP), and plasma plasminogen levels (LP(a)). In total, 9 pQTLs have been associated with 17 disease or physiological phenotypes.

Finally, to investigate whether the pQTLs identified here are associated with VTE or CAD, we examined the results of 2 previously published meta-analyses. The INVENT^30^ study is a large meta-analysis of 7507 cases and 52 632 controls to identify variants associated with VTE. The CARDIoGRAM^29^ study is a large meta-analysis of 22 233 cases and 64 762 controls designed to identify variants associated with CAD, which is predominantly composed of MI. Of 14 common pQTLs, 10 (71.4%) could be tested in the INVENT and CARDIoGRAM datasets (Table XI in the Data Supplement). The KLKB1 pQTL (rs3733402) is significantly associated with VTE; however, this association becomes nonsignificant when the analysis is conditioned on the top 6 SNPs associated with VTE from the literature. The KLKB1 pQTL (rs3733402) is also nominally associated with CAD (*P*=0.0086). The KNG1 pQTL (rs166479) had a nominal *P* value of <0.05 in the INVENT consortium. Although one of the pQTLs for LP(a) (rs41272114) has previously been associated with CAD,^31^ it was not present in either data set. In addition, among the 17 protein biomarkers that we previously identified as being associated with the first MI,^10^ we identified common *cis*-pQTLs for 6 (C3, C3b, KLKB1, LP(a), MMP3, and MMP8) and rare *cis*-pQTLs for 5 (LP(a), MMP8, TAFI, and TIMP4). Although we found pQTLs for these MI biomarkers, they were not associated with CAD in the CARDIoGRAM study, which could indicate that the biomarkers are not causally related to CAD, but may be a result of the relatively small sample size in the GWAS compared with typical Mendelian randomization studies. Thus, although CAD and VTE were not significantly associated with pQTLs, these loci could be used in further larger studies to elucidate functional mechanisms underlying disease.

## Discussion

Using a combination of exome sequencing and exome arrays in 330 individuals, we identified 27 genetic associations between pQTLs and the serum levels of 20 proteins: 14 associations with common variation in *cis*, of which 6 are novel and have not been previously reported; 7 associations with rare variants in *cis*, of which 4 are novel; and 6 associations in *trans*. Ultimately, 15 proteins were associated with single sites and 5 were associated with rare variants. The strongest associations were identified for *cis* variation near the gene locus, but by directly testing the *cis*-pQTLs, we also identified 2 that acted in *trans*. Despite the limitations of our study (including a relatively small sample size and lack of a formal replication cohort), the presence of robust associations suggest that exome analysis is an effective tool to identify genetic variation associated with serum protein levels and that larger sample sizes would likely capture additional *trans* effects.

This is the first study, to the best of our knowledge, that uses exome data to investigate the effects of both common and rare variation on more than 50 protein levels; and thus, it provides insight into rare variant association methods. For rare-variant analysis, we used 3 different methods for grouping variants within a gene and accounted for the additional testing through permutation. Some associations were consistent across all the 3 methods, such as LP(a), which carried a large number of variants (Table IX in the Data Supplement) and for which rare variation has previously been associated with the protein level in the blood.^41^ Others were only significant in 1 test, such as MMP8 when variants were grouped based on CADD score, which could be because of few variants with weak effects and would benefit from larger sample sizes to include more predicted functional sites. Variants with an MAF between 1% and 5% were tested in both the common- and rare-variant analyses. In 2 cases (LP(a) and MMP8), adjusting for the top common pQTL (with an MAF<5%) nullified the association. In addition, for CHIT1, common variation (MAF>5%) was associated with rare variants through cryptic LD and adjusting for the common variant also nullified the association. These data suggest that significant common and rare single sites may drive gene-based rare variant associations.

Of the 14 common pQTLs, 4 are missense variants in the relevant gene. Of the 10 other variants, 3 are intronic, 2 are in the exons of nearby genes, and 5 lie in regions that are predicted to have regulatory functions, such as interrupting protein-binding sites or splicing (Table VIII in the Data Supplement). Analysis of the function of sequences harboring the pQTL can elucidate the mechanism of the variant. For example, it has been shown that rs1801020 in the 3′ untranslated region of the *F12* locus prevents translation of F12.^42^ The mechanisms of the other 4 regulatory pQTLs are not yet understood, but the results shown here point to plausible mechanisms. For instance, ANG and RNASE4 are isoforms of the same gene with different functions and differential expression patterns that are influenced by CTCF.^43^ The ANG pQTL is in the last exon of *RNASE4*, near a CTCF-binding site that affects isoform expression levels.^43^ This, and other potentially regulatory pQTLs, could be functionally tested using in vitro and in vivo assays for changes in gene or isoform expression. Thus, although we focused on exome sequences to generate genotypes for this analysis, imputation enabled us to identify many pQTLs with predicted regulatory effects.

pQTLs can be used to understand the relationship between proteins and disease, either through tracing molecular impacts through pathways or through studies of Mendelian randomization. By examining potential *trans* associations with *cis*-pQTLs, we recapitulated known and recently reported relationships between these proteins. The relationships between F12, kallikrein, and kininogen comprise the start of the intrinsic coagulation pathway,^44^ the association between kallikrein and uPAR has been previously explored,^45^ and the genetic relationship between kallikrein and NTproBNP was identified in a recent GWAS.^37^ We show that kallikrein is able to cleave proBNP in vitro using purified reagents, suggesting that extracellularly, kallikrein could be responsible for cleaving proBNP into NTproBNP and BNP, although further experiments are necessary to verify that this reaction occurs naturally in plasma. We also identified 17 reported disease and physiological phenotype associations with 9 of the pQTLs (8 previously known and 1 novel). Interestingly, 5 of the 6 novel pQTLs were not implicated in GWAS studies. This could reflect a bias in GWAS phenotypes studied or candidate proteins chosen for pQTL studies and supports further work identifying downstream effects of these loci. We observed a nominal association between KLKB1 and CAD, which we previously identified as a biomarker for MI, supporting further examination of this relationship in larger studies. Overall, these findings support the use of pQTLs to identify molecular and phenotypic effects of proteins and help to elucidate underlying mechanisms of disease.

## Appendix

The INVENT Consortium is comprised Philippe Amouyel, Mariza de Andrade, Saonli Basu, Claudine Berr, Jennifer A. Brody, Daniel I. Chasman, Jean-Francois Dartigues, Aaron R. Folsom, Marine Germain, Hugoline de Haan, John Heit, Jeanine Houwing-Duitermaat, Christopher Kabrhel, Peter Kraft, Grégoire Legal, Sara Lindström, Ramin Monajemi, Pierre-Emmanuel Morange, Bruce M. Psaty, Pieter H. Reitsma, Paul M. Ridker, Lynda M. Rose, Frits R. Rosendaal, Noémie Saut, Eline Slagboom, David Smadja, Nicholas L. Smith, Pierre Suchon, Weihong Tang, Kent D. Taylor, David-Alexandre Trégouët, Christophe Tzourio, Marieke C.H. de Visser, Astrid van Hylckama Vlieg, Lu-Chen Weng, and Kerri L. Wiggins.

## Sources of Funding

This work was supported by an independent grant from the K.G. Jebsen Foundation in Norway and partially funded by Tethys Bioscience. T. Solomon is supported by an institutional award to the UCSD Genetics Training Program from the National Institute for General Medical Sciences, T32 GM008666.

## Disclosures

None.

## Supplementary Material

## CLINICAL PERSPECTIVE

Cardiovascular diseases, including coronary artery disease and venous thromboembolism, are the leading cause of death worldwide. Biomarkers are important tools to diagnose or measure risk of disease, but the causal relationship between biomarkers and diseases is often not clear. Genetic variants that affect levels of protein biomarkers could be used to examine causal relationships between biomarkers and diseases and to provide mechanistic insight into disease. In this study, we investigated whether genetic variants were associated with the levels of 51 serum proteins, 17 of which we had previously identified as predictors for myocardial infarction in the Tromsø Study. We analyzed genotype data from exome sequencing and exome arrays and investigated whether common and rare genetic variation located near the gene (*cis*) that coded for each protein was associated with protein levels. We identified 13 proteins associated with common *cis* variants and 7 proteins associated with rare *cis* variation; 8 of these proteins we had previously identified as biomarkers. To identify pathway-level regulation, we tested whether these significantly associated *cis* variants were also associated in *trans* with the levels of the other 50 proteins in this study. We identified that genetic variation affecting the levels of kallikrein, a protease involved in coagulation, also affect the levels of NTproBNP, a known biomarker for heart failure. We experimentally show that kallikrein can cleave proBNP into NTproBNP and BNP. Our study shows that identifying genetic variants that affect protein levels can provide novel insights and expand our knowledge of the mechanisms of disease.
