# Supplementary material for: Associations Between Common and Rare Exonic Genetic Variants and Serum Levels of 20 Cardiovascular-Related Proteins: The Tromsø Study
Source: Circ Cardiovasc Genet. 2016 Aug 16;9(4):375–83. doi: 10.1161/CIRCGENETICS.115.001327 (PMC4982757; doi:10.1161/CIRCGENETICS.115.001327)

SUPPLEMENTAL MATERIAL

Supplemental Methods

Protein Assay

Protein levels were determined using ELISAs and performed by Tethys Biosciences, Inc (Emeryville, CA), as previously reported[1]. Normal Human Serum from VWR (Radnor, PA), a pool made from 10-16% of Tromsø study samples, and dilution buffer were used as controls. Each anti-protein antibody was either directly conjugated to an AlexaFluor 647 or was biotinylated and detected with a streptavidin-conjugated AlexaFluor 647. Each protein underwent 8 serial dilutions. All samples were performed in triplicate. The eight-point standard curve was measured in six replicates per plate. The AlexaFluor 647-labeled antibodies were detected using the Erenna System (Singulex, Inc., Alameda, CA). Emission from each labeled antibody is measured with a photon detector. The photon detector transmits an electronic pulse for each photon detected, and pulses are counted in 1-ms bins. Binned pulses that exceed a six standard deviation threshold above background are counted. Pulses are recorded as photons/minute. For each protein, it must be detected in >70% of samples, there must be more than 2 logs of standard curve linear range in the ELISA, and there was less than 20% of variance between within-plate replicates for the assay to be considered successful.

Variant Identification and Annotation

Genotypes were determined using either the Illumina Infinium HD HumanExome BeadChip (N=87) or whole-exome sequencing (N=243) using Agilent SureSelect 50 Mb or V4 capture kits and Illumina TruSeq paired-end 100bp cluster kits. Sequence reads were mapped to the reference human genome (hg19) using BWA (version 0.7.10-r789) with default parameters and then

processed using Picard (version 1.115, tool Mark Duplicates) (http://broadinstitute.github.io/picard) and GATK (version 3.3-0, tools RealignerTargetCreator, IndelRealigner, BaseRecalibrator, PrintReads, and HaplotypeCaller). We previously showed that the concordance of the exome sequencing and array genotyping data used in this study is 99.33%[2], therefore we felt confident that we could combine the genotypes from both platforms. Using the array data or information from both on and off-target reads[3] from the sequencing data, genotypes were imputed to the whole genome using Beagle (version 4.0, r1398) and haplotypes from unrelated individuals from the European (EUR) and East Asian (EAS) superpopulations of the 1000 Genomes Project Phase 3[4] for sites with a combined MAF >1%. Due to the difference in coverage and imputation quality between sites that were exome sequenced or assayed by array, we only used sites that had a call rate of >90% in their respective datasets, and then added in additional imputation sites passing QC thresholds (allelic r^2 of >0.3). These two datasets were combined to get a final VCF with imputed and genotyped sites for both exome sequenced and exome genotyped individuals. Because the Tromsø Study is a population-based cohort study, it naturally includes some proportion of related individuals. Of the 330 individuals assayed, 20 were related to another individual in the study at an identity-by-descent value of 0.1 for exome sequenced individuals or 0.2 for arrayed individuals, based on genome-wide data.

All significant common variants were annotated for functional effects using variant effect predictor (VEP)[5], RefSeq genes, the hg19 reference genome, GeneVisible (http://genevisible.com/search) and ROADMAP[6] data of the 28-state chromHMM for Liver (E066), HepG2 (E118), and Monocyte (E029) cells. All rare variants (MAF≤5%) were annotated using VEP, RefSeq, and hg19.

Power Calculations

Our power to detect causal variants of varying effect sizes was determined using an equation from the Abecasis laboratory (http://genome.sph.umich.edu/wiki/Power_Calculations:_Quantitative_Traits) for common variants and the SKAT R package[7] for rare variants. Power for the common variant analyses was calculated using a sample size of 300 individuals, a phenotypic variance (R^2) from 0.0 to 1.0, and alpha levels of 6.97×10^{-7} for the *cis* association, 7.29×10^{-5} for the *cis*-acting-in-*trans* analysis, and 1.25×10^{-8} for the *trans* analysis (Supplemental Table 4, Supplemental Figure 1). Power for the rare variant analysis was calculated using a sample size of 300 individuals, an effect size (β in standard deviations) from 0.0 to 5.0, the default haplotypes (European) for the SKAT package, a causal MAF cutoff of 5%, a sampling subregion length of 3kb, and alpha levels of 3.72×10^{-4} for the *cis* association, 5.30×10^{-5} for the *cis*-acting-in-*trans*, and 9.21×10^{-6} for the *trans* associations (Supplemental Table 4, Supplemental Figure 2).

Supplemental References:

1. Wilsgaard T, Mathiesen EB, Patwardhan A, Rowe MW, Schirmer H, Løchen ML, et al. Clinically significant novel biomarkers for prediction of first ever myocardial infarction: the tromsø study. *Circ Cardiovasc Genet*. 2015;8:363-71.

2. Carson AR, Smith EN, Matsui H, Brækkan SK, Jepsen K, Hansen J, et al. Effective filtering strategies to improve data quality from population-based whole exome sequencing studies. *BMC Bioinformatics*. 2014;15:125.

3. Pasaniuc B, Rohland N, McLaren PJ, Garimella K, Zaitlen N, Li H, et al. Extremely low-coverage sequencing and imputation increases power for genome-wide association studies. *Nat Genet*. 2012;44:631-5.

4. Abecasis GR, Auton A, Brooks LD, DePristo MA, Durbin RM, Handsaker RE, et al. An integrated map of genetic variation from 1,092 human genomes. *Nature*. 2012;491:56-65.

5. McLaren W, Pritchard B, Rios D, Chen Y, Flicek P and Cunningham F. Deriving the consequences of genomic variants with the Ensembl API and SNP Effect Predictor. *Bioinformatics*. 2010;26:2069-70.

6. Bernstein BE, Stamatoyannopoulos JA, Costello JF, Ren B, Milosavljevic A, Meissner A, et al. The NIH Roadmap Epigenomics Mapping Consortium. *Nat Biotechnol*. 2010;28:1045-8.

7. Wu MC, Lee S, Cai T, Li Y, Boehnke M and Lin X. Rare-variant association testing for sequencing data with the sequence kernel association test. *Am J Hum Genet*. 2011;89:82-93.

8. Kim S, Swaminathan S, Inlow M, Risacher SL, Nho K, Shen L, et al. Influence of genetic variation on plasma protein levels in older adults using a multi-analyte panel. *PLoS One*. 2013;8:e70269.

9. Johansson Å, Enroth S, Palmblad M, Deelder AM, Bergquist J and Gyllensten U. Identification of genetic variants influencing the human plasma proteome. *Proc Natl Acad Sci U S A*. 2013;110:4673-8.

10. Lourdusamy A, Newhouse S, Lunnon K, Proitsi P, Powell J, Hodges A, et al. Identification of cis-regulatory variation influencing protein abundance levels in human plasma. *Hum Mol Genet*. 2012;21:3719-26.

11. Liu Y, Buil A, Collins BC, Gillet LC, Blum LC, Cheng LY, et al. Quantitative variability of 342 plasma proteins in a human twin population. *Mol Syst Biol*. 2015;11:786.

12. Kyriakou T, Seedorf U, Goel A, Hopewell JC, Clarke R, Watkins H, et al. A common LPA null allele associates with lower lipoprotein(a) levels and coronary artery disease risk. *Arterioscler Thromb Vasc Biol*. 2014;34:2095-9.

13. Zhu C, Odeberg J, Hamsten A and Eriksson P. Allele-specific MMP-3 transcription under in vivo conditions. *Biochem Biophys Res Commun*. 2006;348:1150-6.

Supplemental Table 1: Cohort statistics

	Sex	N	Age	BMI	N Exome Sequenced	N Exome Arrayed
VTE Controls	Females	64	65.29 (50-74)	26.40 (17.6-38.6)	45 (70%)	19 (30%)
	Males	70	64.14 (46-74)	25.85 (20.3-34.6)	52 (74%)	18 (26%)
VTE Cases	Females	100	63.71 (50-75)	27.21 (18.6-41.8)	74 (74%)	26 (26%)
	Males	96	63.45 (45-74)	26.94 (19.7-36.4)	72 (75%)	24 (25%)
**Total**		330	64.03 (45-75)	26.69 (17.6-41.8)	243 (74%)	87 (26%)

N, number

Supplemental Table 2: The fifty-one proteins and 50 loci (C3 and C3b derive from the same gene and locus, but are considered two proteins here) used in this study.

Protein Symbol	Protein Name	Gene	Chr	Locus Start	Locus End	N *cis* Variants	N Individuals Measured	Phenotypic Mean	Range (min-max)	Units
a2-AP	alpha-2-antiplasmin	*SERPINF2*	17	1146129	2158559	1295	303	3.06	0.07-17.79	ug/mL
ACE	angiotensin-converting enzyme	*ACE*	17	61054421	62075741	1075	318	858.45	15.51-3344.92	ng/mL
ADIPOQ	adiponectin	*ADIPOQ*	3	186060462	187076252	766	329	6.81	0.74-99.32	ug/mL
AGER	advanced glycosylation end product-specific receptor	*AGER*	6	31648744	32652099	3523	303	0.40	0.08-1.67	ng/mL
AGT	angiotensinogen	*AGT*	1	230338271	231350336	678	330	1.37	0.01-13.71	ug/mL
ANG	angiogenin	*ANG*	14	20652335	21662345	1516	298	219.78	89.63-482.54	ng/mL
APOA1	apolipoprotein A-1	*APOA1*	11	116206468	117208338	721	302	1582.73	0.08-3704.94	ug/mL
APOB	apolipoprotein B-100	*APOB*	2	20724300	21766945	482	303	19.44	3.82-189.05	ug/mL
APOC3	apolipoprotein C-III	*APOC3*	11	116200623	117203787	715	294	255.52	108.25-960.93	ug/mL
BGLAP	osteocalcin	*BGLAP*	1	155711950	156713123	1564	328	6.27	0.60-63.58	ug/mL
BSG	basigin	*BSG*	19	71324	1083493	2306	328	42.99	15.03-87.72	ng/mL
C3	complement C3	*C3*	19	6177845	7220662	1714	284	233.77	71.76-631.00	mg/mL
C3b	complement component C3b	*C3*	19	6177845	7220662	1714	301	2.95	1.05-16.21	ug/mL
CCL5	C-C motif chemokine 5	*CCL5*	17	33698495	34707377	923	328	155.92	7.77-759.97	ng/mL
CD14	monocyte differentiation antigen CD14	*CD14*	5	139511312	140513286	1104	328	244.71	76.28-739.07	ng/mL
CD163	scavenger receptor cysteine-rich type 1 protein M130	*CD163*	12	7123411	8156414	930	328	180.84	14.71-4863.99	ng/mL
CD40-L	CD40 ligand	*CD40LG*	X	135230335	136242549	441	303	13.31	1.84-154.09	ng/mL
CHIT1	chitotriosidase-1	*CHIT1*	1	202685206	203698860	951	303	57.02	0.27-477.70	ng/mL
CRP	C-reactive protein	*CRP*	1	159182078	160184379	1050	303	199.32	2.50-4877.52	ng/mL
CST3	cystatin-C	*CST3*	20	23114293	24118574	459	302	678.73	239.86-2412.46	ng/mL
CTSG	cathepsin G	*CTSG*	14	24542723	25545466	1539	330	37.26	6.88-186.27	ng/mL
CXCL10	C-X-C motif chemokine 10	*CXCL10*	4	76442268	77444689	985	303	0.05	0.01-0.74	ng/mL
DCN	decorin	*DCN*	12	91039034	92073359	128	303	13.47	6.00-31.13	ng/mL
DPP4	dipeptidyl peptidase 4	*DPP4*	2	162348754	163431052	530	328	992.83	225.56-5879.35	ng/mL
F12	coagulation factor XII	*F12*	5	176329138	177336577	955	303	24.82	0.94-50.27	ug/mL
Fetuin A	alpha-2-HS-glycoprotein	*AHSG*	3	185830849	186839107	752	302	924.06	401.88-3961.18	ug/mL

FTH1	ferritin heavy chain	*FTH1*	11	61231756	62235132	918	329	286.80	6.05-4905.20	ng/mL
HP	haptoglobin	*HP*	16	71588507	72594955	1000	301	799.22	0.06-4090.92	ug/mL
HSPA1B	heat shock 70kDa protein 1B	*HSPA1B*	6	31295511	32298031	3372	303	4.05	0.90-62.02	ng/mL
ICAM1	intercellular adhesion molecule 1	*ICAM1*	19	9881516	10897291	1920	328	34.59	4.31-97.50	ng/mL
KLKB1	plasma kallikrein	*KLKB1*	4	186648671	187679625	798	303	29494.68	2.46-59991.93	ng/mL
KNG1	kininogen-1	*KNG1*	3	185935097	186960678	809	302	84574.02	5.57-302245.75	ng/mL
LBP	lipopolysaccharide-binding protein	*LBP*	20	36474884	37505653	953	303	984.41	170.31-2056.94	ng/mL
LP(a)	apolipoprotein(A)	*APOA*	6	160452514	161587407	1080	303	343.47	12.11-3656.08	ng/mL
MMP3	stromelysin-1	*MMP3*	11	102206527	103214342	1056	328	11.26	2.21-75.17	ng/mL
MMP8	neutrophil collagenase	*MMP8*	11	102082525	103095685	971	302	14.34	1.47-68.09	ng/mL
MMP9	matrix metalloproteinase-9	*MMP9*	20	44137546	45145200	1312	303	458.41	106.99-2665.14	ng/mL
MPO	myeloperoxidase	*MPO*	17	55847216	56858296	1116	303	60.57	12.77-271.17	ng/mL
NTproBNP	n-terminus pro-brain natriuretic protein	*NPPB*	1	11417520	12418992	1380	303	0.30	0.02-6.38	ng/mL
PAI-1	plasminogen activator inhibitor 1	*SERPINE1*	7	100270378	101282547	2129	301	38.30	15.35-95.10	ng/mL
REN	renin	*REN*	1	203623943	204635465	1018	329	0.63	0.05-3.33	ng/mL
SHBG	sex hormone-binding globulin	*SHBG*	17	7017381	8036700	2576	303	1.79	0.21-7.28	ug/mL
TAFI	carboxypeptidase B2	*CPB2*	13	46127321	47179211	498	303	27.92	15.36-68.45	ug/mL
THBS1	thrombospondin-1	*THBS1*	15	39373279	40389668	413	320	44.55	4.05-190.52	ug/mL
THBS4	thrombospondin-4	*THBS4*	5	78831169	79879107	810	303	2.79	0.16-192.66	ug/mL
TIMP1	metalloproteinase inhibitor 1	*TIMP1*	X	46941689	47946190	501	328	61.42	7.27-176.27	ng/mL
TIMP4	metalloproteinase inhibitor 4	*TIMP4*	3	11694567	12700851	573	302	5.20	1.88-18.00	ng/mL
TNFRSF11B	tumor necrosis factor receptor superfamily member 11B	*TNFRSF11B*	8	119435795	120464383	175	328	49.80	11.71-152.77	ng/mL
TNFRSF1B	tumor necrosis factor receptor superfamily member 1B	*TNFRSF1B*	1	11727059	12769277	1355	328	19.79	7.56-49.06	ng/mL
uPAR	urokinase plasminogen activator surface receptor	*PLAUR*	19	43650246	44674498	1362	303	1.73	0.54-4.91	ng/mL
VCAM1	vascular cell adhesion protein 1	*VCAM1*	1	100685195	101704601	326	328	138.01	39.81-679.86	ng/mL

Supplemental Table 3: Number of tests performed for each type of association analysis and the P-value cutoffs using Bonferroni correction or permutations for a FWER < 0.05.

Variant Class	Analysis	Number of tests	Bonferroni cutoff	Permutation cutoff
Common, single site	*Cis*	100,378	4.98×10^{-7}	6.91×10^{-7}
	Cis-acting-in-*trans*	663	7.40×10^{-5}	7.29×10^{-5}
	trans	5,119,278	9.77×10^{-9}	1.25×10^{-8}
Rare, collapsed	*Cis*	153	3.21×10^{-4}	3.72×10^{-4}
	Cis-acting-in-*trans*	918	5.34×10^{-5}	5.30×10^{-5}
	trans	7,803	6.16×10^{-6}	9.21×10^{-6}

Supplemental Table 4: Amount of phenotypic variance explained (R^2) and effect size (β) detected for the various analyses when there is 80% power.

**Variant Class**	**Analysis**	**Alpha**	**Variance Explained (R^2)**
	cis	6.91×10^{-7}	0.113
Common, single-site	*cis*-as-*trans*	7.29×10^{-5}	0.078
	trans	1.25×10^{-8}	0.143
**Variant Class**	**Analysis**	**Alpha**	**Effect Size (β)**
	cis	3.72×10^{-4}	0.80
Rare, collapsed [100% causal]	*cis*-as-*trans*	5.30×10^{-5}	0.90
	trans	9.21×10^{-6}	1.0
	cis	3.72×10^{-4}	1.25
Rare, collapsed [50% causal]	*cis*-as-*trans*	5.30×10^{-5}	1.45
	trans	9.21×10^{-6}	1.75

Supplemental Table 5: Number of variants that were directly genotyped or imputed for the exome sequenced and exome arrayed individuals.

	**Exome Sequenced (N = 243)**	**Exome Arrayed (N = 87)**	**Combined**
**Genotyped**	24,008	2,563	24,915
**Imputed**	129,502	56,195	138,415
**Total**	153,510	58,758	158,137

N, number of individuals

Supplemental Table 6: Type of variants that were directly genotyped or imputed for the exome sequenced and exome arrayed individuals.

Variant type	Exome Sequenced (Genotyped)	Exome Arrayed (Genotyped)	Exome Sequenced (Imputed)	Exome Arrayed (Imputed)	% of total Exome Sequenced variants that were imputed	% of total Exome Arrayed variants that were imputed
Intergenic	1161	355	66432	25886	98.3%	98.6%
Non-coding RNA	694	30	783	470	53.0%	94.0%
Intronic	14700	459	59702	27640	80.2%	98.4%
UTR variant	1323	53	2017	1168	60.4%	95.7%
Synonymous	2488	73	203	600	7.5%	89.2%
Missense	3378	1568	313	380	8.5%	19.5%
Coding sequence indels	192	0	16	34	7.7%	100.0%
start or stop related variants	72	25	7	8	8.9%	24.2%

Supplemental Table 7: Reported disease associations of the significant *cis*-pQTLs.

Locus	Top Variant	Chr	Position	Known pQTL? (if reported direction of effect matches this study's direction of effect)	Schadt eQTL	GTEx eQTL	Genome-wide associations	OMIM
AGT	rs4762	1	230845977	Kim[8] (yes)		AGT sun-exposed skin, transformed fibroblasts, non-exposed skin, subcutaneous adipose, lung, colon, breast, testis, esophagus (2.0x10-6 to 1.3x10-33); RP11-99J16_A.2 transformed fibroblasts, sun-exposed skin, subcutaneous adipose (6.0x10-10 to 1.0x10-14)		in LD with rs699 and rs5051 (r^2 = 0.24, D' = 1) which are associated with hypertension, lower promoter activity and transcription amount
ANG	rs3748338	14	21167576	novel	rs8008440 (r^2=0.24) -log10p = 5.7144			
C3	rs11569415	19	6716279	Johansson[9] (yes)				in LD with rs2230199 (r^2 = 0.98, D' = 0.99) which is associated with age-related macular degeneracy
C3	rs2230199	19	6718387	Johansson[9] (yes)			age-related macular degeneration	age-related macular degeneracy, slow and fast in electrophoresis, kidney production of C3
CHIT1	rs2486951	1	203174921	Lourdusamy[10] (yes)		CHIT1 whole blood (4.2x10-8); ADORA1 transformed fibroblasts (1.4x10-6)		
F12	rs1801020	5	176836532	Liu[11] (yes)		F12 liver (2.3x10-10); MXD3 esophagus (4.2x10-6)	F12 levels, protective effect on acute coronary syndrome in people with stable CAD, activated partial thromplastin time, serum metabolite levels	

KLKB1	rs3733402	4	187158034	novel	F11 artery, esophagus, brain, and muscle (1.8-7.0x10-6)	B-type natriuretic peptide, midregional-proadrenomedullin and C-terminal-pro-endothelin-1, serum metabolite levels	PKD Sedi is two mutations (compound heterozygosity with rs121964952) that reduce binding to HMWK
KNG1	rs166479	3	186443250	novel		activated partial thromboplastin time	
LBP	rs2232613	20	36997655	Lourdusamy[10] (yes)		reduced binding capacity for LPS, homozygous has low serum concentrations, protease cleavage site, carriers have cleaved LBP which doesn't bind LPS and has low cytokine after LPS	
APOA	rs41272114	6	161006077	Kyriakou[12] (yes)		plasma plasminogen levels	
APOA	rs56393506	6	161089307	Novel			
MMP3	rs7926920	11	102698724	Zhu[13] (yes)	MMP1 transformed fibroblasts (6.8x10-9); WTAPP1 transformed fibroblasts, testis (1.9-8.0x10-7)	Serum MMP-1 levels	
MMP8	rs35231465	11	102584135	novel			
SERPINF2	rs2070863	17	1648502	novel	SERPINF2 skeletal muscle, sun-exposed skin, subcutaneous adipose, transformed fibroblasts, esophagus, testis (5.3x10-7 to 8.8x10-18); WRD81 esophagus, sun-exposed skin, breast (2x10-6 to 1.3x10-12)		

Supplemental Table 8: Functional annotations of the 14 significant *cis*-pQTLs using GeneVisble, variant effect predictor (VEP) and ROADMAP data of the 28-state chromHMM for Monocyte (E029), Liver (E066) and HepG2 (E118) cells.

Protein	Gene	Top Variant	Chr	Postion	Expressed in	macrophage ROADMAP	Liver ROADMAP	HepG2 ROADMAP	VEP Annotations
AGT	*AGT*	rs4762	1	230845977	liver	quiescent	downstream promoter TSS 2	Transcribed & regulatory	missense variant (*AGT*); TF binding site variant (Nrsf)
ANG	*ANG*	rs3748338	14	21167576	liver	quiescent	Weak transcription	quiescent	missense (*RNASE4*)
LP(a)	*APOA*	rs41272114	6	161006077	liver	quiescent	quiescent	quiescent	splice donor variant (*APOA*)
LP(a)	*APOA*	rs56393506	6	161089307		quiescent	quiescent	quiescent	upstream gene variant (*APOA*); regulatory region variant (CTCF binding site)
C3	*C3*	rs11569415	19	6716279	liver	quiescent	quiescent	Weak transcription	intronic (*C3*)
C3b	*C3*	rs2230199	19	6718387	liver	quiescent	downstream promoter TSS 2	downstream promoter TSS 2	missense (*C3*); TF binding site variant (Egr1)
CHIT1	*CHIT1*	rs2486951	1	203174921	macrophage	quiescent	quiescent	quiescent	regulatory region variant (promoter flanking region); intergenic
F12	*F12*	rs1801020	5	176836532	liver	quiescent	Poised promoter	Active transcription start site	5' UTR variant (*F12*)
KLKB1	*KLKB1*	rs3733402	4	187158034	liver	quiescent	quiescent	quiescent	missense (*KLKB1*)
KNG1	*KNG1*	rs1656921	3	186442833	liver	quiescent	quiescent	quiescent	intronic (*KNG1*)
LBP	*LBP*	rs2232613	20	36997655	liver	quiescent	Weak transcription	quiescent	missense (*LBP*)
MMP3	*MMP3*	rs2155013	11	102701858	joints	quiescent	quiescent	quiescent	intronic (*WTAPP1*)
MMP8	*MMP8*	rs35231465	11	102584135	bone marrow	quiescent	quiescent	quiescent	Stop gain (*MMP8*); 3' UTR variant (*MMP8*)
a2-AP	*SERPINF2*	rs8077638	17	1640793	liver	Transcribed 3' preferential	Transcribed 3' preferential	Transcribed 3' preferential	synonymous variant (*WDR81*); upstream gene variant; downstream gene variant

Supplemental Table 9: List of rare variants that comprise each significant rare *cis*-pQTL association and their P-values from the single-site associations. An X means that the variant was used in the indicated clustering method. Gray regions mean that the collapsed region was not significant using that clustering method.

Protein	Chr	Start	rsID	Ref/Effect	MAF	Single-site P-value	MAF ≤ 5%	Deleterious	CADD10
**AGER**	6	32147044	rs142802704	AAG/A	0.03477	0.0005711	X		
	6	32147157	rs41268928,rs116420335	G/C	0.03465	0.0005482	X		
	6	32148724	rs41270464,rs114878357	C/T	0.04125	0.8907	X		
	6	32148814	.	GGGTTATACAGGAGAGA/G	0.0022	NA	X		
	6	32148909	rs201575255	CTG/C	0.00165	NA	X		
	6	32149065	rs181811810	C/T	0.00441	NA	X		
	6	32149140	rs3176931	C/T	0.00658	NA	X		
	6	32149471	rs114564020	G/A	0.0022	NA	X		
	6	32149571	.	C/A	0.01322	0.01551	X		
	6	32149801	rs9391855,rs116515025	C/T	0.02211	0.03112	X		
	6	32149883	rs204996,rs116334026	C/T	0.0297	0.1413	X		
	6	32150047	rs77170610	C/T	0.00704	NA	X		
	6	32150107	.	TGAGGCCCTATCTCAGG/T	0.0022	NA	X		
	6	32150303	rs144335694	T/C	0.0022	NA	X		
	6	32150523	.	T/C	0.0022	NA	X		
	6	32150872	.	G/A	0.0022	NA	X		
	6	32151443	rs2070600,rs114177847	C/T	0.0396	0.001871	X		
	6	32151458	rs80096349,rs116828224	G/A	0.00165	NA	X		
	6	32151539	.	G/A	0.0022	NA	X		
	6	32151882	rs115111668	C/T	0.0022	NA	X		
**Fetuin A**	3	186329282	rs111451953	G/A	0.03084	0.4621	X		
	3	186330883	.	G/A	0.00441	NA	X		
	3	186331119	.	T/A	0.00441	NA	X		
	3	186331138	rs150486317	C/T	0.01159	0.5768	X	X	
	3	186331245	rs190631595	G/T	0.0022	NA	X		
	3	186331298	.	A/C	0.00231	NA	X		
	3	186331299	.	G/C	0.00231	NA	X		
	3	186333378	.	C/A	0.0022	NA	X		
	3	186334343	rs79747711	T/G	0.00441	NA	X		
	3	186334932	.	A/G	0.0022	NA	X		
	3	186335056	rs140827890	G/A	0.00661	0.5564	X	X	X
	3	186335248	rs144616056	G/A	0.01762	0.5311	X		
	3	186337746	rs149819140	T/C	0.0022	NA	X		
	3	186337871	rs184392275	G/A	0.0022	NA	X		
	3	186338320	.	T/G	0.0022	NA	X		
	3	186338540	rs35799453	T/C	0.00441	NA	X		

	3	186338564	rs35457250	C/T	0.01821	1.48E-06	X	X	X
	3	186338869	rs11540663	C/T	0.01542	0.9813	X		
**CD40LG**	X	135741275	.	G/A	0.0022	NA		X	
	X	135741443	rs148594123	G/A	0.0165	0.001338		X	
**CHIT1**	1	203183825	rs2015402	G/A	0.04846	0.2873	X		
	1	203184018	rs946849	T/C	0.04846	0.2873	X		
	1	203184924	rs80241012	G/A	0.02643	0.1151	X		
	1	203185118	rs17532442	C/T	0.03744	0.2581	X		
	1	203186420	.	A/C	0.01106	0.667	X		
	1	203186666	rs41308417	C/G	0.02212	0.6183	X		
	1	203188379	.	CCCACTGGTTGTCCCGGAAGA TGTAGGGCA/C	0.00661	0.02734	X		
	1	203189093	rs76499133	C/T	0.03111	0.02956	X		
	1	203189350	rs74969659	G/A	0.03304	0.2992	X		
	1	203189634	rs2486959	A/G	0.04626	3.46E-13	X		
	1	203191527	rs56152830	C/T	0.01322	0.4565	X		
	1	203191994	rs7512820	G/A	0.00889	0.3586	X		
	1	203192424	rs181385947	G/A	0.02643	0.9288	X		
	1	203192518	.	G/A	0.00661	0.9764	X		
	1	203193134	rs2486068	T/G	0.04405	1.32E-09	X		
	1	203194544	rs140940634	C/CATT	0.03965	0.5655	X		
	1	203194548	rs184545416	G/T	0.03965	0.5655	X		
	1	203194688	.	C/T	0.00441	NA	X		
	1	203194834	rs137852607	C/T	0.0033	NA	X		
	1	203195006	rs116389839	G/A	0.01322	0.2128	X		
	1	203195126	rs185483258	C/T	0.00229	NA	X		
	1	203195398	rs10920587	C/A	0.00881	0.9725	X		
	1	203195689	rs2486070	G/A	0.04405	9.21E-11	X		
	1	203196479	rs2486071	A/C	0.04626	9.39E-11	X		
	1	203196842	rs56035601	C/T	0.03965	0.5655	X		
	1	203198596	rs72739588	G/A	0.02477	0.2938	X		
**LP(a)**	6	160952621	rs41266381	A/C	0.00441	NA	X		
	6	160952667	rs73012273	C/G	0.01982	0.7021	X		
	6	160952780	rs186413938	C/T	0.0033	NA	X	X	X
	6	160952816	rs41267807	T/C	0.0132	0.4979	X	X	X
	6	160953642	rs41267809	A/G	0.0198	0.01013	X	X	
	6	160960892	.	AAG/A	0.0022	NA	X		
	6	160961137	rs3798220	T/C	0.00828	0.1252	X	X	X
	6	160962115	.	A/G	0.0022	NA	X		
	6	160962151	.	T/G	0.0022	NA	X	X	X
	6	160962185	.	A/G	0.00441	NA	X		
	6	160962190	.	C/A	0.0022	NA	X	X	X
	6	160962366	.	TC/T	0.02535	0.8887	X		
	6	160962368	rs116039216	G/T	0.02546	0.8848	X		
	6	160962370	rs116089584	G/T	0.02804	0.9047	X		

6	160963576	rs41265940	T/A	0.00221	NA	X		
6	160963648	rs6920765	G/A	0.0022	NA	X		
6	160963964	rs41265934	C/G	0.00441	NA	X		
6	160964135	rs41265930	T/C	0.00661	0.868	X		
6	160966559	rs139145675	G/A	0.00165	NA	X	X	X
6	160968863	rs149574804	C/T	0.01982	0.4082	X		
6	160968968	rs41264848	G/A	0.0022	NA	X		
6	160969075	rs41264844	C/T	0.03084	0.5789	X		
6	160969096	rs4708871	C/T	0.02423	0.2038	X		
6	160969113	rs145989243	G/A	0.0022	NA	X		
6	160971286	rs62441900	C/G	0.03965	0.2143	X		
6	160973905	rs62441901	C/G	0.03965	0.2143	X		
6	160976914	.	CA/C	0.00221	NA	X		
6	160978270	rs184372256	A/G	0.00442	NA	X		
6	160978686	rs149526393	A/G	0.00441	NA	X		
6	160985107	rs145783310	A/G	0.0495	0.7805	X		
6	160985438	rs79563112	C/T	0.0495	0.7805	X		
6	160985526	rs118039278	G/A	0.02632	0.0406	X		
6	160997118	rs74617384	A/T	0.02632	0.0406	X		
6	160998052	rs76602267	G/A	0.00441	NA	X		
6	160998143	.	C/T	0.0022	NA	X		
6	161005610	rs55730499	C/T	0.02632	0.0406	X		
6	161005898	.	C/T	0.00221	NA	X		
6	161005908	rs41272116	ACTT/A	0.00221	NA	X		
6	161006077	rs41272114*	C/T	0.0297	1.05E-08	X	X	X
6	161006084	rs76144756	G/A	0.00441	NA	X	X	X
6	161006105	rs41272112	C/T	0.00165	NA	X	X	
6	161007647	.	G/A	0.0022	NA	X		
6	161010118	rs10455872	A/G	0.01815	0.005497	X		
6	161010546	rs41267815	G/C	0.00441	NA	X		
6	161011907	rs74334585	C/T	0.01101	0.1663	X		
6	161012262	rs144958108	A/G	0.00667	0.8523	X		
6	161015301	rs41271036	A/G	0.01101	4.23E-06	X		
6	161016414	.	A/G	0.0022	NA	X		
6	161020526	rs41270998	A/G	0.0022	NA	X		
6	161020532	.	G/A	0.00661	0.417	X		X
6	161021800	.	G/T	0.00221	NA	X		
6	161022107	rs41259144	C/T	0.00662	0.003408	X	X	X
6	161022108	rs186072375	G/T	0.0022	NA	X		X
6	161025782	.	G/T	0.00455	NA	X		
6	161026197	rs117174672	G/A	0.03084	0.2908	X		
6	161026250	.	G/A	0.0022	NA	X		
6	161027256	.	A/G	0.00231	NA	X		
6	161027287	.	G/C	0.00221	NA	X		
6	161027430	rs144587038	G/T	0.01542	0.1217	X		

	6	161027821	rs75055004	A/T	0.0022	NA	X		
	6	161027895	.	TA/T	0.00457	NA	X		
	6	161032267	rs117949336	C/T	0.00461	NA	X		
	6	161032369	rs151298886	C/T	0.00221	NA	X		
	6	161032401	rs78893353	G/A	0.01106	0.1991	X		
	6	161032413	.	G/C	0.00221	NA	X		
	6	161032497	rs112092923	A/G	0.02212	0.09362	X		
	6	161055824	.	C/A	0.00243	NA	X		
	6	161055876	.	C/T	0.00444	NA	X		
	6	161055880	.	G/A	0.00222	NA	X		
	6	161055942	.	G/C	0.00441	NA	X		
	6	161055968	.	C/A	0.0022	NA	X		
	6	161055991	.	C/T	0.0022	NA	X		
	6	161056129	.	T/TAGA	0.0022	NA	X		
	6	161071476	rs200491482	T/G	0.00165	NA	X	X	X
	6	161071615	.	G/A	0.0022	NA	X		
	6	161071638	.	T/C	0.0022	NA	X		
	6	161087336	rs117643720	T/C	0.0022	NA	X		
	6	161087368	.	G/A	0.00881	0.06095	X		
	6	161087372	rs181060240	T/C	0.01322	0.03437	X		
**MMP8**	11	102584135	rs35231465*	G/A	0.03642	6.86E-07			X
	11	102585130	.	A/G	0.00224	NA			X
	11	102586142	rs61753779	A/G	0.00166	NA			X
	11	102592160	rs11602288	G/A	0.00661	0.7785			X
	11	102593266	rs112188995	C/T	0.00993	0.1649			X
**TAFI**	13	46627762	rs145067962	A/T	0.0066	0.003747		X	
	13	46648069	rs140446990	T/C	0.00495	0.0407		X	
	13	46656619	.	C/T	0.0022	NA		X	
**TIMP4**	3	12195137	.	G/C	0.00221	NA		X	X
	3	12195660	rs140022692	G/A	0.01106	0.1126			X
	3	12198889	.	A/G	0.00221	NA		X	X
	3	12200201	.	C/T	0.00221	NA		X	X
	3	12200210	.	G/A	0.00442	NA		X	X
	3	12200219	.	A/G	0.00221	NA		X	X
	3	12200269	.	C/T	0.00221	NA			X

* = variant was tested in both the common analysis and in the rare analysis.

Supplemental Table 10: Pearson's correlation between the proteins that were identified in the trans-pQTL analysis.

	**F12**	**KLKB1**	**KNG1**	**NTproBNP**	**uPAR**
**F12**	1.0	-0.18	0.09	-0.11	-0.06
**KLKB1**	-0.18	1.0	0.29	0.16	0.20
**KNG1**	0.09	0.29	1.0	0.09	0.32
**NTproBNP**	-0.11	0.16	0.09	1.0	0.28
**uPAR**	-0.06	0.20	0.32	0.28	1.0

Supplemental Table 11: Lookup of common *cis*-pQTLs for their associations in the CARDIoGRAM and INVENT meta-analyses.

Protein	Variant	Chr	Start	Ref/Alt	CARDIoGRAM Coronary Artery Disease			INVENT Venous Thromboembolism		
					**P-value**	**β**	**SE**	**P-value**	**β**	**SE**
a2-AP	rs8077638*	17	1640793	C/T	0.3399	0.156	0.017	Not in INVENT		
a2-AP	rs8065251*	17	1637458	G/A	0.3552	0.015	0.017	0.3298	-0.028	0.029
AGT	rs4762	1	230845977	G/A	0.5018	-0.143	0.021	0.1694	-0.048	0.035
ANG	rs3748338	14	21167576	A/T	0.4549	0.020	0.027	0.9519	0.002	0.039
C3/C3b	rs2230199†	19	6718387	G/C	0.1045	0.050	0.031	0.7668	0.010	0.034
CHIT1	rs2486951	1	203174921	A/G	0.7288	-0.006	0.017	0.8562	-0.005	0.028
F12	rs1801020	5	176836532	A/G	0.5115	-0.016	0.246	0.6957	0.011	0.028
**KLKB1‡**	**rs3733402**	4	187158034	G/A	**0.0086**	0.040	0.015	**8.2×10^{-12}**	-0.159	0.023
**KNG1**	**rs166479**	3	186443250	T/C	0.1692	0.020	0.014	**0.0436**	-0.046	0.023
LBP	rs2232613	20	36997655	C/T	0.3624	0.025	0.028	0.3531	0.050	0.054
MMP3	rs7926920	11	102698724	G/A	0.1016	0.023	0.014	0.7347	0.008	0.023

Bolded indicates a nominal P-value of <0.05. β, effect size. SE, standard error
* rs8077638 was not present in the INVENT dataset, so the next most significant variant (rs8065251) was looked up in both studies as well.
† The top variant for C3 (rs11569415) was not present in either CARDIoGRAM or INVENT so the next most significant variant (rs2230199) was used.
‡ The association with VTE was no longer significant after adjustment that included rs4253417.
No significant variants for LP(a) or MMP8 were present in either study.

Supplemental Figure 1: Power to detect common variation pQTLs with varying effect sizes in the three stages of analysis. A) Power curve for *cis*-pQTLs using the permutation cutoff of 6.91×10^{-7} as the alpha. B) Power curve for testing the *cis*-pQTLs acting-in-*trans* using the permutation cutoff of 7.29×10^{-5} as the alpha. C) Power curve for *trans*-pQTLs using the permutation cutoff of 1.25×10^{-8}. The x-axis is measuring the amount of variance of the phenotype that a variant explains (R^2).

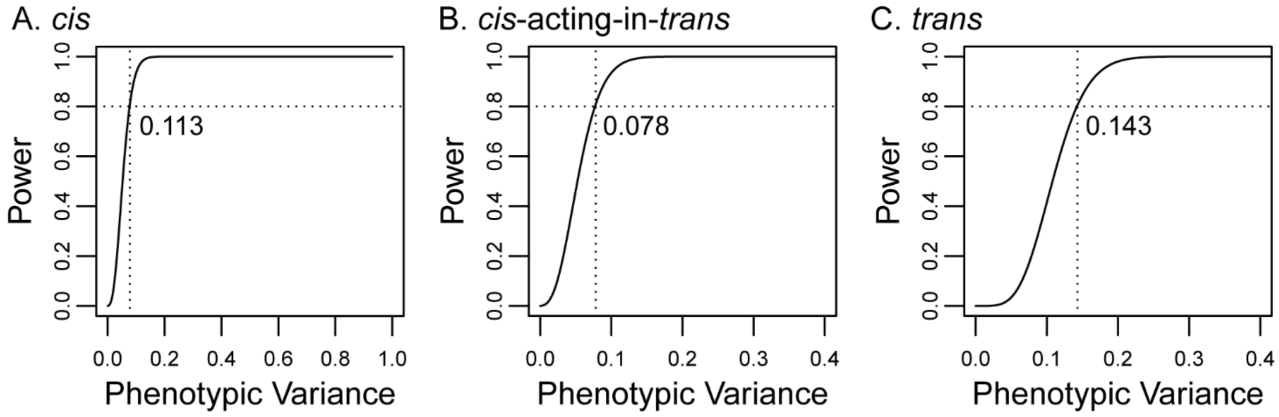

Supplemental Figure 2: Power to detect rare variation pQTLs with varying effect sizes in the three stages of analysis. Effect size is measured in standard deviations (β). The top row assumes that all variants have an equal effect and that all variants are causal. The bottom row assumes that all variants have an equal effect and that half of the variants tested are causal. A) power to detect *cis* associations, alpha = 3.72 x 10^{-4}; B) power to detect *cis*-acting-in-*trans* pQTLs, alpha = 5.30 x 10^{-5}; C) power to detect *trans* associations, alpha = 9.21 x 10^{-6}. The x-axis is measuring the effect size (β) in standard deviations.

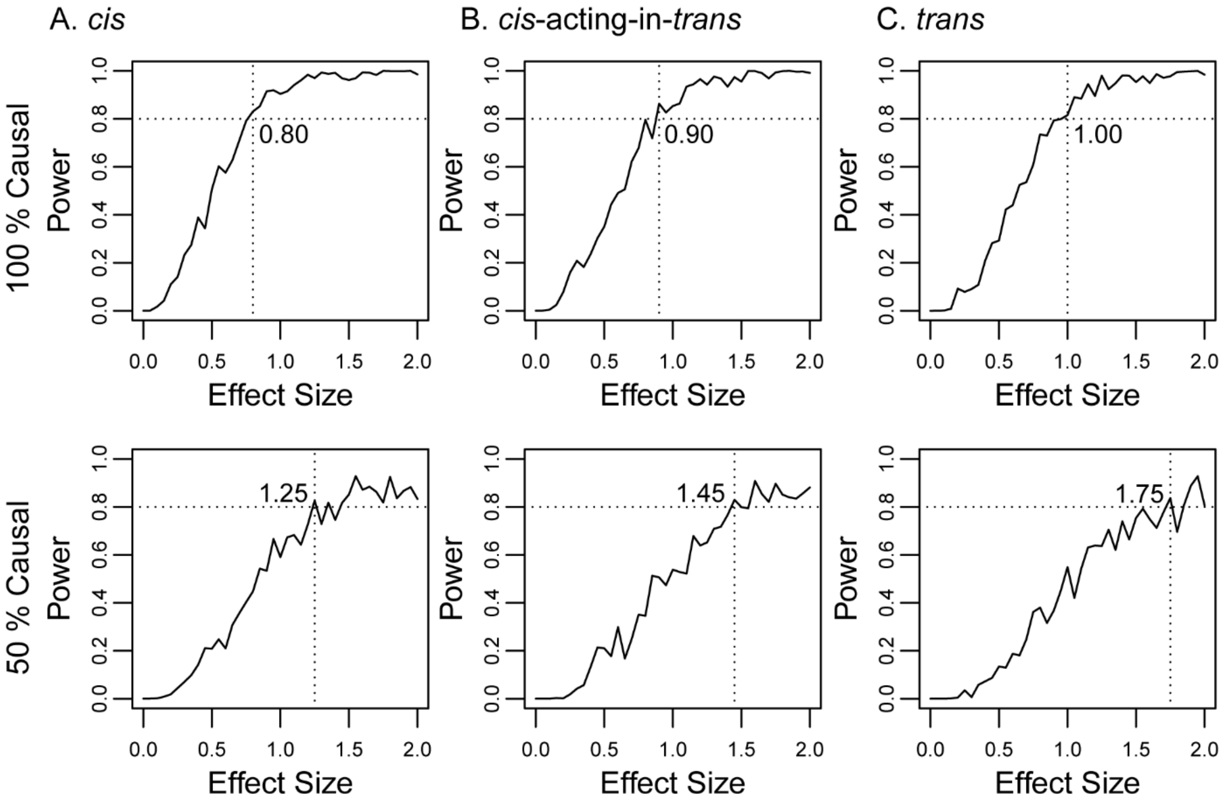

Supplemental Figure 3: Pearson's correlation of the protein levels. Dendrogram shows clustering based on correlation. Black lines are outlining the proteins identified in the *cis*-acting-in-*trans* analysis: F12, KLKB1, KNG1, NTproBNP, uPAR; corresponding values can be found in Supplemental Table 10.

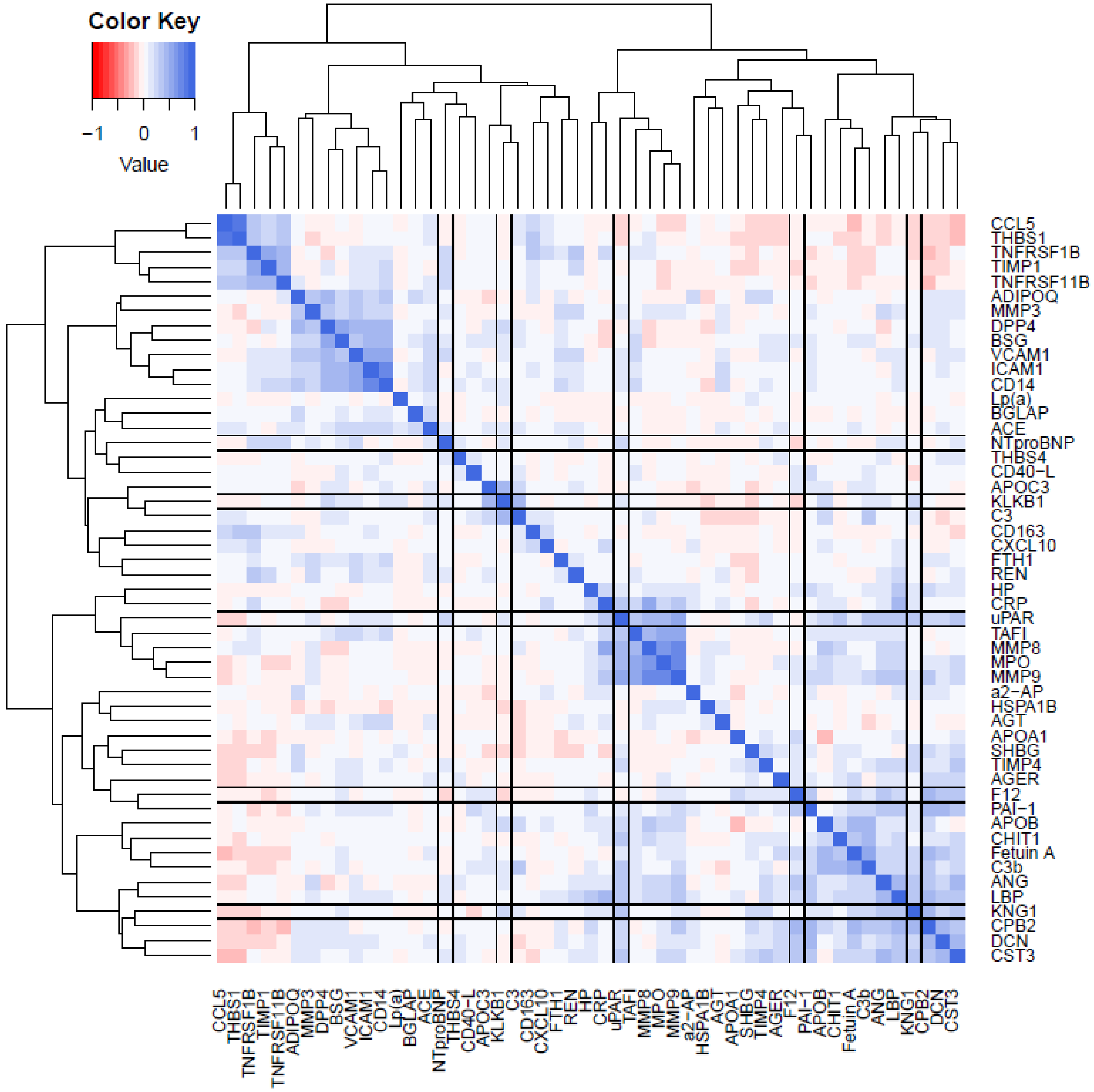

Supplemental Figure 4: Silver stain of proBNP incubated for 1 hour with varying concentrations of kallikrein, with and without a kallikrein-specific inhibitor, PPACK II. Kallikrein concentrations are 74.8nM, 374nM, 748nM, and 1497nM. The upper bands are the light and heavy chains of kallikrein. The lower band is proBNP. 374nM of kallikrein was chosen to perform the silver stain and western blot in Figure 4 of the paper.

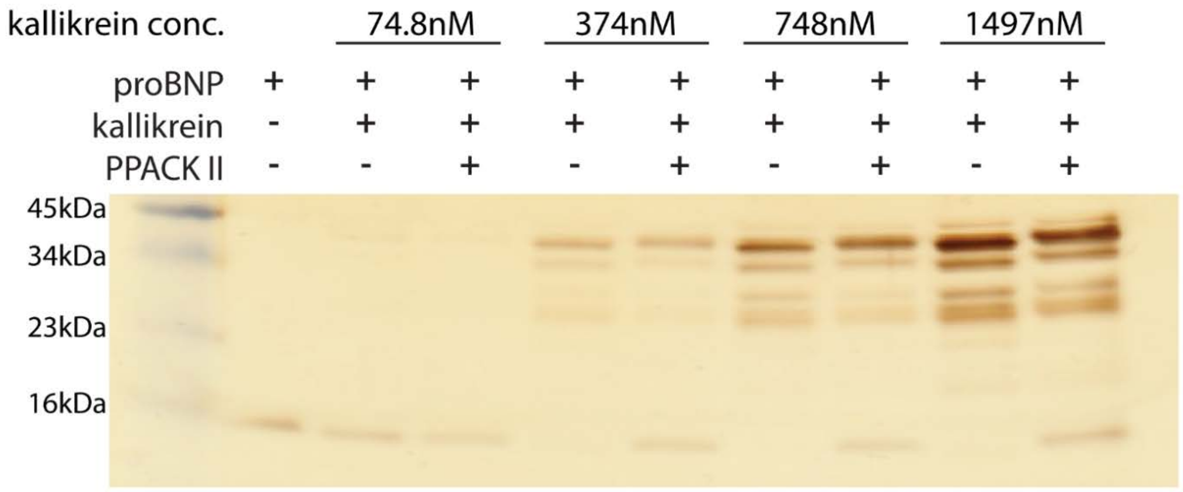

Supplement: Supplementary file 1 [file hcg-9-375-s001.pdf]
