# Supplementary material for: Associations Between Common and Rare Exonic Genetic Variants and Serum Levels of 20 Cardiovascular-Related Proteins: The Tromsø Study
Source: Circ Cardiovasc Genet. 2016 Aug 16;9(4):375–83. doi: 10.1161/CIRCGENETICS.115.001327 (PMC4982757; doi:10.1161/CIRCGENETICS.115.001327)
Supplement: Supplementary file 2 [file hcg-9-375-s002.pdf]

DOI: 10.1161/CIRCGENETICS.115.001327

Associations Between Common and Rare Exonic Genetic Variants and Serum Levels of Twenty Cardiovascular-Related Proteins: The Tromsø Study

Running title: *Solomon et al.; Common and rare cardiovascular-related pQTLs*

Terry Solomon, BS[1]; Erin N. Smith, PhD[2]; Hiroko Matsui, MS[2]; Sigrid K. Braekkan, PhD[3,4]; INVENT consortium; Tom Wilsgaard, PhD[5]; Inger Njølstad, MD, PhD[3,5]; Ellisiv B. Mathiesen, MD, PhD[3,6]; John-Bjarne Hansen, MD, PhD[3,4]; Kelly A. Frazer, PhD[2,3,7]

[1]Biomedical Sciences Graduate Program, [2]Department of Pediatrics & Rady's Children's Hospital, [7]Institute for Genomic Medicine, University of California San Diego, La Jolla, CA; [3]K.G. Jebsen Thrombosis Research and Expertise Centre (TREC), Department of Clinical Medicine, UiT The Arctic University of Norway, [4]Division of Internal Medicine, University Hospital of North Norway, [5]Department of Community Medicine, UiT The Arctic University of Norway, [6]Brain and Circulation Research Group, Department of Clinical Medicine, UiT The Arctic University of Norway, Tromsø, Norway

Circulation
Cardiovascular Genetics

Correspondence:
Kelly A. Frazer, PhD
University of California, San Diego
Frazer Lab, Institute for Genomic Medicine
9500 Gilman Drive #0761
La Jolla, CA 92093-0761
Tel: 858-246-0208
Fax: 858-246-1818
E-mail: kafrazer@ucsd.edu

Journal Subject Terms: Genetic, Association Studies; Biomarkers; Atherosclerosis; Thrombosis

Abstract:

Background - Genetic variation can be used to study causal relationships between biomarkers and diseases. Here, we identify new common and rare genetic variants associated with cardiovascular-related protein levels (protein quantitative trait loci, pQTLs). We functionally annotate these pQTLs, predict and experimentally confirm a novel molecular interaction and determine which pQTLs are associated with diseases and physiological phenotypes.
Methods and Results - As part of a larger case/control study of VTE, serum levels of 51 proteins implicated in cardiovascular diseases were measured in 330 individuals from the Tromsø Study. Exonic genetic variation near each protein's respective gene (*cis*) was identified using sequencing and arrays. Using single site and gene-based tests, we identified 27 genetic associations between pQTLs and the serum levels of 20 proteins: 14 associated with common variation in *cis*, of which six are novel (i.e. not previously reported); seven associations with rare variants in *cis*, of which four are novel; and six associations in *trans*. Of the 20 proteins, 15 were associated with single sites and seven with rare variants. *cis*-pQTLs for kallikrein and F12 also show *trans* associations for proteins (uPAR, kininogen) known to be cleaved by kallikrein as well as with NTproBNP. We experimentally demonstrate that kallikrein can cleave proBNP (NTproBNP precursor) *in vitro*. Nine of the pQTLs have previously identified associations with 17 diseases and/or physiological phenotypes.
Conclusions - We have identified *cis* and *trans* genetic variation associated with the serum levels of 20 proteins and utilized these pQTLs to study molecular mechanisms underlying diseases and/or physiological phenotypes.

American Heart Association
Circulation
Cardiovascular Genetics

Key words: venous thromboembolism; coronary artery disease; biomarker; exome; cardiovascular genomics; candidate genes, protein, human, NT-proBNP

DOI: 10.1161/CIRCGENETICS.115.001327

Introduction

Recent advances in genetics have yielded an unprecedented number of loci associated with disease and are beginning to yield mechanistic insight, such as with the IRX3/5 association with BMI, which revealed brown adipose as an important regulator of body weight[1]. Genetic variation underlying molecular phenotypes, such as proteins and transcript expression levels, can be important tools in constructing the effects of genetic variations into pathways, ultimately resulting in physiological understanding of diseases[2]. Protein levels in particular may be more informative for understanding disease because there is often a poor correlation between transcript and protein levels[3]. Several prior studies[4-6] have systematically identified genetic variations associated with protein levels and isoforms (protein quantitative trait loci or pQTLs). While most studies have focused on common variation (minor allele frequency ≥5%), rare variants, which can show strong loss of function effects, can be useful in understanding causality and pinpointing drug targets, such as deletion mutations in *PSCK9* that abolish the PSCK9 protein and reduce LDL cholesterol levels[7]. Systematic screening for rare variation influencing a wide variety of proteins, however, has not yet been performed.

American Heart Association

Circulation Cardiovascular Genetics

Genetic variation is also useful in identifying causal relationships between biomarkers and diseases using tools such as Mendelian randomization[8] and could be used to ascertain how risk factors differentially affect various diseases, as well as trace causal pathways between risk loci and disease. We are investigating risk factors for cardiovascular diseases, including myocardial infarction (MI) and venous thromboembolism (VTE) in the Tromsø Study[9], a longitudinal prospective cohort study. We previously assayed 51 cardiovascular-related proteins in 419 first-ever MI cases and 398 controls in serum collected years prior to the MI event[10]. Of the proteins measured, 17 were predictors for MI when considered individually after adjusting

for traditional risk factors. Genetic variation associated with these protein levels could be used to study underlying mechanisms of cardiovascular diseases.

Here, using whole exome sequencing data and HumanCoreExome BeadChips, we investigate if genetic variants are associated with the serum levels of the same 51 cardiovascular-related proteins in 330 individuals chosen from the Tromsø Study because they did or did not go on to develop VTE during the 18 years of follow-up (mean time to VTE of 9 years). The serum samples were collected at study entry enabling us to identify pQTLs associated with baseline protein levels. We perform both common and rare variation association analyses in order to identify *cis*-pQTLs. Further characterization of the *cis*-pQTLs to determine if they also act as *trans*-pQTLs with any of the other 51 cardiovascular-related proteins, recapitulated well-established physiological relationships between F12, kallikrein, uPAR, kininogen, and a recent genetic association with NTproBNP. We experimentally confirmed an inferred physiological interaction from the *trans*-pQTLs by showing that kallikrein cleaves proBNP *in vitro*. We then examine genetic associations from genome-wide association studies on coronary artery disease (CAD) and VTE as well as published literature to identify physiological and disease associations.

American Heart Association. Circulation Cardiovascular Genetics

Methods

The Tromsø Study

The Tromsø Study is a prospective, single-site, cohort study of the inhabitants of Tromsø, Norway. In 1994-1995, 27,158 individuals filled out epidemiological surveys and donated (non-fasting) blood to the National CONOR Biobank[9]. These individuals were followed until 2013, with repeated surveys and identified in national registries that report various diseases and causes of death. In 2013, we identified individuals who between 1995 and 2013 had had an incident of VTE or death due to VTE, regardless of other comorbidities. We chose age and sex-matched

controls randomly from the cohort. These samples were chosen for a currently ongoing case/control study of VTE. DNA and protein levels were ascertained from the blood collected in 1994.

For this specific study, blood and non-fasting serum samples were collected from 330 healthy individuals (166 males, 164 females) aged 45-75 (Supplemental Table 1). There were 196 individuals diagnosed with VTE between study entry (1994-'95) and the eighteen-year follow-up period (2013) and 134 controls without development of VTE during this period. Aspirin usage and other medication information were not collected for the Tromsø study. DNA was isolated from the blood for genotyping and serum samples were used to assay protein levels. The regional committee for medical and health research ethics in North Norway approved the study, and all participants gave informed written consent.

American Heart Association. Circulation Cardiovascular Genetics

Protein Quantification

Protein levels were quantified using the same methods and at the same time as our previous MI study[10], but the samples from people that went on to develop VTE were not included in that study. Briefly, the literature was searched to create a list of over 900 cardiovascular-related proteins that might be potential biomarkers for myocardial infarction and atherosclerosis. This list was then prioritized to 165 candidate proteins, of which 51 had sufficient commercially available reagents (two antibodies and purified protein for control) in order for Tethys Bioscience, Inc (Emeryville, CA) to perform successful sandwich ELISAs (Supplemental Methods, Supplemental Table 2). All protein levels were quantile normalized and mapped to the normal distribution using qnorm in R and significance was tested using Z-scores.

Variant Identification and Annotation

Genotypes were determined using exome sequencing (N=243) or exome genotyping arrays

(N=87). Sequences were mapped and called using BWA[11] and GATK[12], imputed to the 1000 Genomes Project[13] using Beagle[14], and functionally annotated for predicted effect and regulatory regions (see Supplemental Methods).

Statistical Analysis

Associations were performed using EPACTS software[15]. We used sex, age at study entry, BMI at study entry, genotyping platform, and VTE case/control status as covariates. Three covariates (age at serum collection, sex and BMI at serum collection) were associated respectively with ten, ten, and thirteen of the phenotypes (the 51 protein serum levels) when performing linear regressions, defined as having an FDR-adjusted P-value <0.05, and were included for consistency.

American Heart Association. Circulation Cardiovascular Genetics

For common variants (MAF≥1%) we used EMMAX[16] (a mixed model implemented in the EPACTS software package[15]), using q.emmax to test for single-site association. For *cis* associations we included any imputed common variants located within the interval surrounding and including the gene (+/- 500kb from transcript start and stop positions) that encodes the protein(s) being tested (C3 and C3b share the same locus). For *cis*-acting-in-*trans* associations we tested all significantly associated common *cis* variants against each of the other 50 phenotypes. For *trans* associations we tested the 100,378 common variants found in the 50 intervals against each of the 51 phenotypes (Figure 1).

SKAT-O[17] was used to test clusters of rare variants (MAF≤5%) for association as implemented in EPACTS, using the skat-o version of the mmskat test. Rare variants were classified in three ways: 1) MAF≤5%: all rare variants located within the gene body and 2kb upstream; 2) Deleterious: all rare variants located in the gene body and the 2kb upstream region that were annotated as stop-gain, stop-loss, start-loss, essential splice site disruption, frame-shift

causing, or nonsynonymous using VEP annotations; and 3) CADD-score: all rare variants in the gene or the 2kb upstream region with a PHRED-scaled c-score >10, as determined by Kircher *et al.*[18].

We corrected for multiple testing by permuting the phenotype-genotype relationship 1000 times and for each permutation performing all variant-phenotype tests for each association type separately (e.g. *cis*, *cis*-acting-in-*trans*, or *trans*)[19]. We obtained the lowest P-value from each permutation across all phenotypes and created a null distribution of minimum P-values. An association was considered significant (family-wise $P<0.05$) if the nominal P-value was smaller than 95% of the null distribution (Supplemental Table 3).

To test for multiple, independent variants in the same locus, the top variant was included as a covariate until there was no longer a significant association (family-wise $P<0.05$) detected for that protein.

Power Calculations

We calculated power using an equation from the Abecasis laboratory (http://genome.sph.umich.edu/wiki/Power_Calculations:_Quantitative_Traits) for common variants and the SKAT R package[20] for rare variants. We had 80% power to detect effects (R^2) down to 0.113 for the *cis*, common variant analysis and effects (beta) of 1.25 for the *cis*, rare variant analysis (assuming that 50% of the variants are causal), which is comparable to other pQTL studies[4-6, 21, 22]. Further details are in the supplement (Supplemental Methods, Supplemental Figures 1 and 2, Supplemental Table 4).

Clinical and molecular phenotype association

Significant pQTLs from this study were queried against the eQTLs found by Schadt *et al.*[23] in liver cells and the GTEx database[24] (version 4, build 200, accessed at

http://www.gtexportal.org/home/) for all tissue types. Additionally, we determined if they (or a variant in LD) overlapped any of the variants identified as pQTLs in five similarly-sized independent studies that investigated protein levels in serum[4] or plasma[4-6, 22, 25]. We examined pQTLs for clinical significance by determining if the variant has been previously identified and submitted to OMIM[26], the GWAS Catalog[27], or GRASP v2.0[28]. We identified pQTLs that were also significant in large meta-analyses of individuals of European descent for CAD or VTE. Data on CAD was downloaded from www.cardiogramplusc4d.org. For this analysis, we only used the CARDIoGRAM GWAS results[29], as these individuals are of European descent. Data on VTE was shared by the INVENT consortium[30].

In Vitro Assay of proBNP Cleavage

We obtained native kallikrein from human plasma from EMD-Millipore (Darmstadt, Germany; cat no. 420307); recombinant proBNP from Abcam (Cambridge, Ma; cat no. ab151881); the kallikrein inhibitor, PPACK II, from Santa Cruz Biotechnology (Dallas, Tx; cat no. sc-203215). 354ng (374 nM) of kallikrein was incubated with 80ng (606 nM) of proBNP with and without 26.5ng (36.4 μM) of PPACK II for 30min, 60min, and 90min at 37°C. The reactions were stopped by adding 4X LDS sample buffer and DTT, and heating them for 2min at 85°C. The proteins were run on a Tricine-SDS-page gel from ThermoFisher (Waltham, Ma), and either detected using the SilverQuest™ Silver Staining Kit from ThermoFisher (Waltham, Ma) or transferred to a PVDF membrane and detected using an anti-BNP antibody from Novus Biologicals (Littleton, Co; cat no. NB100-62133) and chemiluminescence.

American Heart Association. Circulation Cardiovascular Genetics

Results

Study overview

The subjects were chosen as a sub-study from an ongoing case-control study examining the

genetics of VTE, and include 196 individuals that developed VTE during the 18 year follow-up and 134 individuals that did not (Supplemental Table 1). Serum was assayed for the levels of 51 proteins using ELISAs (Supplemental Table 2). On average, we obtained high quality protein measurements for 311 individuals per phenotype. We investigated if any of the protein levels were associated with VTE case/control status and found no significant associations. Knowing that the protein levels weren't statistically associated with VTE enabled us to combine the VTE cases and controls in order to explore the effects of genetic variation on baseline protein levels.

We performed high coverage (~100X) exome sequencing on DNA from blood samples for 243 individuals and assayed an additional 87 with HumanCoreExome Beadchips. We identified 158,137 variants (direct genotyping and imputation) in the 50 intervals that encode the 51 proteins (Supplemental Table 5). The majority of imputed variants were intergenic or intronic because these were variants not already captured by the genotyping array or were outside of the exome-sequencing target regions (Supplemental Table 6). There was an average of 1,122 variants per locus with the *AGER* locus having the most variants (3,523) and the *CD40LG* locus having the fewest (441) (Supplemental Table 2).

American Heart Association. Circulation Cardiovascular Genetics

Identifying *cis*-pQTLs from common variants

To identify genetic variation associated with serum protein levels, we tested for association between variants within the gene's *cis* locus and the normalized protein level for each of the 51 protein levels, adjusting for sample relatedness and population structure using a kinship matrix and including age, sex, BMI, genotype platform, and subsequent VTE status as covariates. Because of the high likelihood of linkage disequilibrium at the *cis* loci and slight correlations among protein levels, we accounted for multiple testing by performing permutations to obtain a family-wise error rate. We identified significant associations (adjusted P<0.05, nominal

$P<6.97x10^{-7}$) (Table 1, Figure 2) for thirteen of the 51 phenotypes. To test for multiple, independent associations we performed sequential conditioning on the most highly associated variant, and found two independent *cis* associations for LP(a). Of the fourteen *cis*-pQTLs that we report, we have replicated eight known pQTLs and identified six novel pQTLs. The same variant or a variant in LD (r^2>0.5 in EUR) has been previously reported for eight proteins with the same direction of effect that we found: AGT[22], C3[5], C3b[5], CHIT1[6], F12[6, 25], LBP[6], one of the variants for LP(a)[31], and MMP3[32] (Supplemental Table 7). Of the six novel pQTLs that we identified, four proteins have not previously been reported to have a *cis*-pQTL (a2-AP, ANG, KLKB1, and MMP8) and two proteins have been previously associated with a pQTL, but the variant identified here is not in LD with the previous variant (KNG1[25,6] and LP(a)[31]). rs3373402 in *KLKB1* was previously reported to affect KLKB1 binding with kininogen (KNG1) but not affect KLKB1 levels in plasma[33]; therefore while this variant has been previously functionally characterized this is a novel pQTL. We annotated the 14 pQTLs for functional effects and identified their chromatin state in the tissue that they are most highly expressed in (Supplemental Table 8). Ten of the thirteen proteins are predominantly secreted by the liver. Five of the top variants are missense variants, three are in the UTR regions and five lie in predicted regulatory regions based on chromatin state annotations. These analyses suggest possible mechanisms of action for some of the *cis*-pQTLs.

American Heart Association. Circulation Cardiovascular Genetics

Identifying *cis*-pQTLs from rare variation

We next tested whether the combination of multiple rare variants at each *cis*-locus was associated with protein levels. There were 3,675 rare variants identified across all 50 loci. For rare variation association analyses, rare variants are grouped according to frequency or function and then jointly tested for association. Because functional prediction methods vary and it is

DOI: 10.1161/CIRCGENETICS.115.001327

currently unknown what method is superior[34], we used three different classifications (MAF, Deleterious, and CADD-score – see Methods). Across all loci there was a range of 1 to 90 variants used for each method, with the MAF method having the most rare variants and CADD scores having the fewest. To account for multiple testing, we tested all three classifications in each round of permutations to determine the family-wise error rate P-value cutoff. We performed a SKAT-O association test using the same covariates as for the common variant association. We identified eight *cis*-pQTLs that were significant using one or more classifications (adjusted $P<0.05$, nominal $P<3.72\times10^{-4}$) (Table 2, Supplemental Table 9). Of these, *cis* rare variation has been associated with AGER[35], Fetuin A[36], and LP(a) levels[31]; to our knowledge the other five associations are novel.

American Heart Association. Circulation Cardiovascular Genetics

Of the eight proteins associated with rare variation, three were also associated with a common pQTL (CHIT1, LP(a), and MMP8). For LP(a) and MMP8, a common pQTL (with a MAF<5%) was also present on the list of rare variants and removal of these from the rare variant analysis made the rare association non-significant (CADD nominal P-value 0.148 and 0.469, respectively). For CHIT1, the common pQTL had a MAF of 18% and although not on the list of rare variants, when we included this variant as a covariate in the rare variant analysis the association was nullified (nominal P-value 0.147). These results suggest that the rare variants in the *CHIT1* locus were associated with CHIT1 serum levels due to linkage disequilibrium with the common pQTL. Because the driving variant was common, we do not consider the CHIT1 association to be valid, resulting in seven proteins associated with rare variants.

Identifying *trans*-pQTLs

To characterize potential downstream effects of *cis*-pQTLs, we investigated whether any of the common *cis*-pQTLs might also have *trans* effects (*cis*-acting-in-*trans*) on any of the other 50

protein levels. After permutation to obtain adjusted P-values, we identified two *cis*-acting-in-*trans* loci, each of which was significantly associated with three proteins (adjusted P<0.05, nominal P<7.29x10^{-5}) (Table 3). There was significant overlap in the proteins associated with the two loci and the associations were consistent with known physiological relationships between F12, KLKB1, KNG1, and uPAR, and the recently reported genetic relationship with NTproBNP[37] (Figure 3), despite none of the protein levels being strongly correlated (Supplemental Figure 3 and Supplemental Table 10). We did not observe an association between the *cis*-pQTL for *KLKB1* and F12 protein levels despite the known physiological relationships of KLKB1 and F12 (Figure 3). Importantly, the genetic associations of *KLKB1* and *F12* with NTproBNP suggest that KLKB1 may physiologically cleave proBNP (the NTproBNP precursor). These findings illustrate how genetic variation can be used to identify potentially novel physiological relationships among proteins.

We further performed a full pairwise association (*trans*) between any of the variants located in the 50 regions encoding the proteins used in this study and all 51 protein levels. After permutation adjusting (adjusted P<0.05, nominal P<1.25x10^{-8}) we did not find any additional *trans* associations and none of the *cis*-acting-in-*trans* associations remained significant; however 11 of the 14 *cis* associations remained significant.

Using a similar approach to the common variants, we tested if any of the rare variant *cis*-pQTLs were associated with any of the other 50 protein levels and did not observe any significant associations (adjusted P<0.05, nominal P<5.30x10^{-5}). Additionally, we tested all 50 *cis* regions against all 51 protein levels in a pairwise manner, but did not identify additional associations (adjusted P<0.05, nominal P<9.21x10^{-6}), although four of the eight rare *cis* associations were still significant at the more stringent threshold.

American Heart Association.
Circulation
Cardiovascular Genetics

DOI: 10.1161/CIRCGENETICS.115.001327

The role of kallikrein in proBNP maturation

We experimentally tested the *cis*-acting-in-*trans* associations suggesting that kallikrein (KLKB1) may physiologically cleave proBNP. ProBNP is produced as a pro-peptide that may be cleaved intracellularly into BNP and NTproBNP, two biomarkers for heart failure[38], before being secreted by cardiomyocytes in response to cardiac stress. Intracellularly, it is thought that furin or corin cleave proBNP[39], but it is unclear which enzyme cleaves proBNP extracellularly when it is secreted intact[40]. To test whether kallikrein can cleave proBNP *in vitro,* we incubated increasing concentrations of kallikrein (74.8nM, 374nM, 748nM, and 1497nM) with proBNP for 1 hour at body temperature (37°C) and saw progressive depletion of proBNP levels (Supplemental Figure 4). This depletion was prevented with the addition of PPACK II, a kallikrein-specific inhibitor. From this, we chose to incubate 374 nM of kallikrein with proBNP for 30, 60 or 90 minutes and again, we saw that the levels of proBNP decreased (Figure 4). These results suggest that kallikrein has the ability to cleave proBNP *in vivo*.

American Heart Association
Circulation
Cardiovascular Genetics

Annotation of pQTLs using existing databases and GWAS

We investigated whether the 14 common pQTLs that we identified were previously associated with gene expression levels (Supplemental Table 7) using eQTLs from the GTEx database[24] as well as Schadt *et al.*[23] to include additional data from liver samples, as many of the proteins studied are expressed in liver. In the GTEx database, the AGT pQTL was identified as an eQTL in ten tissues (P-values from of $2.0x10^{-6}$ to $1.3x10^{-33}$), the CHIT1 pQTL is an eQTL in whole blood (P-value $4.2x10^{-8}$), the F12 pQTL is an eQTL in liver (P-value $2.3x10^{-10}$) and the pQTL in the *SERPINF2* locus (a2-AP protein) is an eQTL in six tissues (P-values from $5.3x10^{-7}$ to $8.8x10^{-18}$). Additionally, the pQTLs for a2-AP, AGT, CHIT1, F12, KLKB1, and MMP3 were also identified as eQTLs for other nearby genes. In the Schadt dataset rs3748338 in the *ANG* locus is

in LD (r^2=0.24) with an eQTL for ANG (rs8008440). Thus, of the 14 common pQTLs, two have previously been identified as an eQTL for the *cis* gene, three as an eQTL for both the *cis* gene and other nearby genes, and three as an eQTL for nearby gene(s).

We also looked up whether there are any known disease associations with the fourteen pQTLs that we identified using the GWAS catalog[27]. GRASP[28], and OMIM[26] (Supplemental Table 7). The 8 known pQTLs along with the kallikrein pQTL are associated with a variety of phenotypes, including age-related macular degeneration (C3b), activated partial thromboplastin times (F12), serum metabolites (KLKB1), binding of LBP to LPS (LBP), and plasma plasminogen levels (LP(a)). In total, nine pQTLs (eight known and KLKB1) have been associated with 17 disease and/or physiological phenotypes.

American Heart Association.
Circulation
Cardiovascular Genetics

Finally, to investigate if the pQTLs identified here are associated with VTE or CAD, we examined the results of two previously published meta-analyses. The INVENT[30] study is a large meta-analysis of 7,507 cases and 52,632 controls to identify variants associated with VTE. The CARDIoGRAM[29] study is a large meta-analysis of 22,233 cases and 64,762 controls designed to identify variants associated with CAD, which is predominantly comprised of MI. Of 14 common pQTLs, ten (71.4%) could be tested in the INVENT and CARDIoGRAM datasets (Supplemental Table 11). The KLKB1 pQTL (rs3733402) is significantly associated with VTE; however this association becomes non-significant when the analysis is conditioned on the top six SNPs associated with VTE from the literature. The KLKB1 pQTL (rs3733402) is also nominally associated with CAD (P=0.0086). The KNG1 pQTL (rs166479) had a nominal P-value <0.05 in the INVENT consortium. While one of the pQTLs for LP(a) (rs41272114) has previously been associated with CAD[31], it was not present in either dataset. Additionally, among the 17 protein biomarkers that we previously identified as being associated with first MI[10], we identified

common *cis*-pQTLs for six (C3, C3b, KLKB1, LP(a), MMP3, MMP8) and rare *cis*-pQTLs for five (LP(a), MMP8, TAFI, and TIMP4). While we found pQTLs for these MI biomarkers, they weren't associated with CAD in the CARDIoGRAM study, which could indicate that the biomarkers are not causally related to CAD, but may also be a result of the relatively small sample size in the GWAS compared to typical Mendelian randomization studies. Thus, while CAD and VTE were not significantly associated with pQTLs, these loci could be used in further larger studies to elucidate functional mechanisms underlying disease.

Discussion

Using a combination of exome sequencing and exome arrays in 330 individuals, we identified 27 genetic associations between pQTLs and the serum levels of 20 proteins: 14 associated with common variation in *cis*, of which six are novel and have not been previously reported; seven associations with rare variants in *cis*, of which four are novel; and six associations in *trans*. Ultimately, 15 proteins were associated with single sites and seven were associated with rare variants. The strongest associations were identified for *cis* variation near the gene locus, but by directly testing the *cis*-pQTLs, we also identified two that acted in *trans*. Despite the limitations of our study (including a relatively small sample size and lack of a formal replication cohort) the presence of robust associations suggest that exome analysis is an effective tool to identify genetic variation associated with serum protein levels and that larger sample sizes would likely capture additional *trans* effects.

This is the first study, to the best of our knowledge, that uses exome data to investigate the effects of both common and rare variation on more than 50 protein levels and thus, provides insight into rare-variant association methods. For rare-variant analysis we used three different methods for grouping variants within a gene and accounted for the additional testing through

permutation. Some associations were consistent across all three methods, such as LP(a), which carried a large number of variants (Supplemental Table 9) and for which rare variation has previously been associated with the protein level in the blood[41]. Others were only significant in one test, such as MMP8 when variants were grouped based on CADD score, which could be due to few variants with weak effects and would benefit from larger sample sizes to include more predicted functional sites. Variants with a MAF between 1% and 5% were tested in both the common and rare variant analyses. In two cases (LP(a) and MMP8) adjusting for the top common pQTL (with a MAF<5%) nullified the association. Additionally, for CHIT1, common variation (MAF>5%) was associated with rare variants through cryptic LD and adjusting for the common variant also nullified the association. These data suggest that significant common and rare single sites may drive gene-based rare-variant associations.

American Heart Association. Circulation Cardiovascular Genetics

Of the fourteen common pQTLs, four are missense variants in the relevant gene. Of the ten other variants, three are intronic, two are in the exons of nearby genes, and five lie in regions that are predicted to have regulatory functions, such as interrupting protein-binding sites or splicing (Supplemental Table 8). Analysis of the function of sequences harboring the pQTL can elucidate the mechanism of the variant. For example, it has been shown that rs1801020 in the 3' UTR of the *F12* locus prevents translation of F12[42]. The mechanisms of the other four regulatory pQTLs are not yet understood, but the results shown here point to plausible mechanisms. For instance, *ANG* and *RNASE4* are isoforms of the same gene with different functions and differential expression patterns that are influenced by CTCF[43]. The ANG pQTL is in the last exon of *RNASE4*, near a CTCF binding site which affects isoform expression levels[43]. This, and other potentially regulatory pQTLs, could be functionally tested using *in vitro* and *in vivo* assays for changes in gene or isoform expression. Thus, although we focused on exome sequences to

generate genotypes for this analysis, imputation enabled us to identify many pQTLs with predicted regulatory effects.

pQTLs can be used to understand the relationship between proteins and disease, either through tracing molecular impacts through pathways or through studies of Mendelian randomization. By examining potential *trans* associations with *cis*-pQTLs, we recapitulated known and recently reported relationships between these proteins. The relationships between F12, kallikrein, and kininogen comprise the start of the intrinsic coagulation pathway[44], the association between kallikrein and uPAR has been previously explored[45], and the genetic relationship between kallikrein and NTproBNP was identified in a recent GWAS[37]. We show that kallikrein is able to cleave proBNP *in vitro* using purified reagents, suggesting that extracellularly, kallikrein could be responsible for cleaving proBNP into NTproBNP and BNP, although further experiments are necessary to verify that this reaction occurs naturally in plasma. We also identified 17 reported disease and physiological phenotype associations with nine of the pQTLs (eight previously known and one novel). Interestingly, five of the six novel pQTLs were not implicated in GWAS studies. This could reflect a bias in GWAS phenotypes studied or candidate proteins chosen for pQTL studies and supports further work identifying downstream effects of these loci. We observed a nominal association between KLKB1 and CAD, which we previously identified as a biomarker for MI, supporting further examination of this relationship in larger studies. Overall, these findings support the use of pQTLs to identify molecular and phenotypic effects of proteins and help to elucidate underlying mechanisms of disease.

American Heart Association
Circulation
Cardiovascular Genetics

Appendix: The INVENT Consortium is comprised of Philippe Amouyel, Mariza de Andrade, Saonli Basu, Claudine Berr, Jennifer A Brody, Daniel I Chasman, Jean-Francois Dartigues, Aaron R Folsom, Marine Germain, Hugoline de Haan, John Heit, Jeanine Houwing-Duitermaat,

Christopher Kabrhel, Peter Kraft, Grégoire Legal, Sara Lindström, Ramin Monajemi, Pierre-Emmanuel Morange, Bruce M Psaty, Pieter H Reitsma, Paul M Ridker, Lynda M Rose, Frits R Rosendaal, Noémie Saut, Eline Slagboom, David Smadja, Nicholas L Smith, Pierre Suchon, Weihong Tang, Kent D Taylor, David-Alexandre Trégouët, Christophe Tzourio, Marieke CH de Visser, Astrid van Hylckama Vlieg, Lu-Chen Weng, and Kerri L Wiggins.

Sources of Funding: This work was supported by an independent grant from the K.G. Jebsen Foundation in Norway and partially funded by Tethys Bioscience. TS is supported by an institutional award to the UCSD Genetics Training Program from the National Institute for General Medical Sciences, T32 GM008666.

Disclosures: None.

American Heart Association
Circulation
Cardiovascular Genetics

References:

1. Claussnitzer M, Dankel SN, Kim KH, Quon G, Meuleman W, Haugen C, et al. FTO obesity variant circuitry and adipocyte browning in humans. *N Engl J Med*. 2015;373:895-907.

2. Schadt EE. Molecular networks as sensors and drivers of common human diseases. *Nature.* 2009;461:218-223.

3. Anderson L, Seilhamer J. A comparison of selected mRNA and protein abundances in human liver. *Electrophoresis*. 1997;18:533-537.

4. Melzer D, Perry JR, Hernandez D, Corsi AM, Stevens K, Rafferty I, et al. A genome-wide association study identifies protein quantitative trait loci (pQTLs). *PLoS Genet.* 2008;4:e1000072.

5. Johansson Å, Enroth S, Palmblad M, Deelder AM, Bergquist J, Gyllensten U. Identification of genetic variants influencing the human plasma proteome. *Proc Natl Acad Sci U S A*. 2013;110:4673-4678.

6. Lourdusamy A, Newhouse S, Lunnon K, Proitsi P, Powell J, Hodges A, et al. Identification of cis-regulatory variation influencing protein abundance levels in human plasma. *Hum Mol Genet.* 2012;21:3719-3726.

7. Cohen JC, Boerwinkle E, Mosley TH, Hobbs HH. Sequence variations in PCSK9, low LDL, and protection against coronary heart disease. *N Engl J Med*. 2006;354:1264-1272.

8. Lawlor DA, Harbord RM, Sterne JA, Timpson N and Davey Smith G. Mendelian randomization: using genes as instruments for making causal inferences in epidemiology. *Stat Med*. 2008;27:1133-1163.

9. Jacobsen BK, Eggen AE, Mathiesen EB, Wilsgaard T, Njølstad I. Cohort profile: the Tromso Study. *Int J Epidemiol*. 2012;41:961-967.

10. Wilsgaard T, Mathiesen EB, Patwardhan A, Rowe MW, Schirmer H, Løchen ML, et al. Clinically significant novel biomarkers for prediction of first ever myocardial infarction: the Tromsø Study. *Circ Cardiovasc Genet*. 2015;8:363-371.

11. Li H, Durbin R. Fast and accurate short read alignment with Burrows-Wheeler transform. *Bioinformatics*. 2009;25:1754-1760.

12. Van der Auwera GA, Carneiro MO, Hartl C, Poplin R, Del Angel G, Levy-Moonshine A, et al. From FastQ data to high confidence variant calls: the Genome Analysis Toolkit best practices pipeline. *Curr Protoc Bioinformatics*. 2013;11:11.10.1-11.10.33.

13. Abecasis GR, Auton A, Brooks LD, DePristo MA, Durbin RM, Handsaker RE, et al. An integrated map of genetic variation from 1,092 human genomes. *Nature*. 2012;491:56-65.

14. Browning BL, Browning SR. Genotype Imputation with millions of reference samples. *Am J Hum Genet.* 2016;98:116-126.

15. EPACTS [computer program]. Version 3.2.5: University of Michigan Center for Statistical Genetics; 2014.

16. Kang HM, Sul JH, Service SK, Zaitlen NA, Kong SY, Freimer NB, et al. Variance component model to account for sample structure in genome-wide association studies. *Nat Genet*. 2010;42:348-354.

17. Lee S, Wu MC, Lin X. Optimal tests for rare variant effects in sequencing association studies. *Biostatistics.* 2012;13:762-775.

18. Kircher M, Witten DM, Jain P, O'Roak BJ, Cooper GM, Shendure J. A general framework for estimating the relative pathogenicity of human genetic variants. *Nat Genet*. 2014;46:310-315.

19. Hirschhorn JN, Daly MJ. Genome-wide association studies for common diseases and complex traits. *Nat Rev Genet.* 2005;6:95-108.

20. Wu MC, Lee S, Cai T, Li Y, Boehnke M, Lin X. Rare-variant association testing for sequencing data with the sequence kernel association test. *Am J Hum Genet*. 2011;89:82-93.

21. Garge N, Pan H, Rowland MD, Cargile BJ, Zhang X, Cooley PC, et al. Identification of quantitative trait loci underlying proteome variation in human lymphoblastoid cells. *Mol Cell Proteomics*. 2010;9:1383-1399.

American Heart Association
Circulation Cardiovascular Genetics

22. Kim S, Swaminathan S, Inlow M, Risacher SL, Nho K, Shen L, et al. Influence of genetic variation on plasma protein levels in older adults using a multi-analyte panel. *PLoS One.* 2013;8:e70269.

23. Schadt EE, Molony C, Chudin E, Hao K, Yang X, Lum PY, et al. Mapping the genetic architecture of gene expression in human liver. *PLoS Biol.* 2008;6:e107.

24. Consortium G. The Genotype-Tissue Expression (GTEx) project. *Nat Genet*. 2013;45:580-585.

25. Liu Y, Buil A, Collins BC, Gillet LC, Blum LC, Cheng LY, et al. Quantitative variability of 342 plasma proteins in a human twin population. *Mol Syst Biol*. 2015;11:786.

26. Hamosh A, Scott AF, Amberger JS, Bocchini CA, McKusick VA. Online Mendelian Inheritance in Man (OMIM), a knowledgebase of human genes and genetic disorders. *Nucleic Acids Res*. 2005;33:D514-517.

27. Welter D, MacArthur J, Morales J, Burdett T, Hall P, Junkins H, et al. The NHGRI GWAS Catalog, a curated resource of SNP-trait associations. *Nucleic Acids Res.* 2014;42:D1001-1006.

28. Eicher JD, Landowski C, Stackhouse B, Sloan A, Chen W, Jensen N, et al. GRASP v2.0: an update on the Genome-Wide Repository of Associations between SNPs and phenotypes. *Nucleic Acids Res*. 2015;43:D799-804.

29. Schunkert H, König IR, Kathiresan S, Reilly MP, Assimes TL, Holm H, et al. Large-scale association analysis identifies 13 new susceptibility loci for coronary artery disease. *Nat Genet*. 2011;43:333-338.

30. Germain M, Chasman DI, de Haan H, Tang W, Lindström S, Weng LC, et al. Meta-analysis of 65,734 individuals identifies TSPAN15 and SLC44A2 as two susceptibility loci for venous thromboembolism. *Am J Hum Genet*. 2015;96:532-542.

31. Kyriakou T, Seedorf U, Goel A, Hopewell JC, Clarke R, Watkins H, et al. A common LPA null allele associates with lower lipoprotein(a) levels and coronary artery disease risk. *Arterioscler Thromb Vasc Biol*. 2014;34:2095-2099.

32. Zhu C, Odeberg J, Hamsten A, Eriksson P. Allele-specific MMP-3 transcription under in vivo conditions. *Biochem Biophys Res Commun*. 2006;348:1150-1156.

33. Katsuda I, Maruyama F, Ezaki K, Sawamura T, Ichihara Y. A new type of plasma prekallikrein deficiency associated with homozygosity for Gly104Arg and Asn124Ser in apple domain 2 of the heavy-chain region. *Eur J Haematol*. 2007;79:59-68.

34. Santorico SA, Hendricks AE. Progress in methods for rare variant association. *BMC Gene*t. 2016;17 Suppl 2:6.

Circulation Cardiovascular Genetics

35. Hudson BI, Carter AM, Harja E, Kalea AZ, Arriero M, Yang H, et al. Identification, classification, and expression of RAGE gene splice variants. *FASEB J*. 2008;22:1572-1580.

36. Yuasa I, Umetsu K. Genetic polymorphism of human alpha 2HS-glycoprotein: characterization and application to forensic hemogenetics. *Electrophoresis*. 1988;9:404-410.

37. Musani SK, Fox ER, Kraja A, Bidulescu A, Lieb W, Lin H, et al. Genome-wide association analysis of plasma B-type natriuretic peptide in blacks: the Jackson Heart Study. Circ Cardiovasc Genet. 2015;8:122-130.

38. Clerico A, Fontana M, Zyw L, Passino C, Emdin M. Comparison of the diagnostic accuracy of brain natriuretic peptide (BNP) and the N-terminal part of the propeptide of BNP immunoassays in chronic and acute heart failure: a systematic review. *Clin Chem*. 2007;53:813-822.

39. Semenov AG, Tamm NN, Seferian KR, Postnikov AB, Karpova NS, Serebryanaya DV, et al. Processing of pro-B-type natriuretic peptide: furin and corin as candidate convertases. *Clin Chem.* 2010;56:1166-1176.

40. Tonne JM, Campbell JM, Cataliotti A, Ohmine S, Thatava T, Sakuma T, et al. Secretion of glycosylated pro-B-type natriuretic peptide from normal cardiomyocytes. *Clin Chem*. 2011;57:864-873.

41. Clarke R, Peden JF, Hopewell JC, Kyriakou T, Goel A, Heath SC, et al. Genetic variants associated with Lp(a) lipoprotein level and coronary disease. *N Engl J Med*. 2009;361:2518-2528.

42. Kanaji T, Okamura T, Osaki K, Kuroiwa M, Shimoda K, Hamasaki N, et al. A common genetic polymorphism (46 C to T substitution) in the 5'-untranslated region of the coagulation factor XII gene is associated with low translation efficiency and decrease in plasma factor XII level. *Blood*. 1998;91:2010-2014.

43. Sheng J, Luo C, Jiang Y, Hinds PW, Xu Z, Hu GF. Transcription of angiogenin and ribonuclease 4 is regulated by RNA polymerase III elements and a CCCTC binding factor (CTCF)-dependent intragenic chromatin loop. *J Biol Chem*. 2014;289:12520-12534.

44. Bhoola KD, Figueroa CD, Worthy K. Bioregulation of kinins: kallikreins, kininogens, and kininases. *Pharmacol Rev*. 1992;44:1-80.

45. Portelli MA, Siedlinski M, Stewart CE, Postma DS, Nieuwenhuis MA, Vonk JM, et al. Genome-wide protein QTL mapping identifies human plasma kallikrein as a post-translational regulator of serum uPAR levels. *FASEB J*. 2014;28:923-934.

American Heart Association
Circulation
Cardiovascular Genetics

Table 1: Significant *cis*-pQTLs from the common-variant association analysis

Protein	Gene	Top Variant	Chr (b37)	Position (b37)	Alleles (Ref/Alt)	Alt Allele Frequency	Nominal P-value	Adjusted P-value	β	R^2
a2-AP	*SERPINF2*	rs8077638	17	1640793	C/T	0.19	$5.4x10^{-37}$	<0.001	-1.15	0.42
AGT	*AGT*	rs4762	1	230845977	G/A	0.14	$4.4x10^{-22}$	<0.001	1.08	0.25
ANG	*ANG*	rs3748338	14	21167576	A/T	0.11	$1.9x10^{-12}$	<0.001	0.86	0.16
C3	*C3*	rs11569415	19	6716279	G/A	0.15	$6.9x10^{-10}$	<0.001	-0.63	0.13
C3B	*C3B*	rs2230199	19	6718387	G/C	0.23	$1.2x10^{-12}$	<0.001	-0.65	0.16
CHIT1	*CHIT1*	rs2486951	1	203174921	A/G	0.18	$3.7x10^{-21}$	<0.001	-1.01	0.26
F12	*F12*	rs1801020	5	176836532	A/G	0.76	$2.5x10^{-32}$	<0.001	0.99	0.38
KLKB1	*KLKB1*	rs3733402	4	187158034	G/A	0.53	$4.4x10^{-12}$	<0.001	-0.51	0.15
KNG1	*KNG1*	rs166479	3	186443250	T/C	0.41	$1.7x10^{-10}$	<0.001	-0.46	0.13
LBP	*LBP*	rs2232613	20	36997655	C/T	0.10	$2.2x10^{-22}$	<0.001	-1.20	0.27
LP(a)*	*APOA*	rs41272114	6	161006077	C/T	0.030	$3.1x10^{-8}$	0.002	-1.27	0.10
LP(a)*	*APOA*	rs56393506	6	161089307	C/T	0.083	$1.7x10^{-7}$	0.011	0.66	0.08
MMP3	*MMP3*	rs7926920	11	102698724	G/A	0.35	$2.4x10^{-14}$	<0.001	-0.41	0.17
MMP8	*MMP8*	rs35231465	11	102584135	G/A	0.036	$1.9x10^{-7}$	0.012	-1.10	0.09

* LP(a) has two independent *cis*-pQTLs. rs56393506 was identified as an independent pQTL for LP(a) by performing the association analysis using genotypes from the top variant (rs41272114) as a covariate.
Ref, reference; Alt, alternate; β, effect size of association in standard deviation units per each copy of the alternate allele; and R^2, amount of phenotypic variation explained by the variant.

Circulation Cardiovascular Genetics American Heart Association.

DOI: 10.1161/CIRCGENETICS.115.001327

Table 2: Rare-variant *cis*-pQTLs that are significant using at least one of the three grouping methods

Protein	MAF≤5%		Deleterious		CADD10	
	Nominal P-value	Adjusted P-value	Nominal P-value	Adjusted P-value	Nominal P-value	Adjusted P-value
AGER	$3.2x10^{-4}$	0.041	0.003	n.s.	0.006	n.s.
CD40L	0.042	n.s.	$9.8x10^{-5}$	0.009	0.003	n.s.
CHIT1*	$4.3x10^{-8}$	<0.001	0.108	n.s.	0.127	n.s.
Fetuin A	$2.5x10^{-4}$	0.026	$1.5x10^{-5}$	0.002	$2.4x10^{-6}$	<0.001
LP(a)	$1.1x10^{-5}$	0.002	$2.6x10^{-8}$	<0.001	$4.4x10^{-7}$	<0.001
MMP8	0.247	n.s.	$7.7x10^{-4}$	n.s.	$6.3x10^{-6}$	0.002
TAFI	0.014	n.s.	$5.2x10^{-5}$	0.003	0.002	n.s.
TIMP4	0.050	n.s.	$2.4x10^{-4}$	0.026	$1.7x10^{-4}$	0.018

* Not significant after adjusting for the common pQTL (rs2486951).
n.s., not significant.

American Heart Association
Circulation Cardiovascular Genetics

DOI: 10.1161/CIRCGENETICS.115.001327

Table 3: *Cis*-pQTLs that also act as *trans*-pQTLs

Variant	Protein	Nominal P-value	Adjusted P-value	β	R^2
rs1801020 in the *F12* locus	F12	$2.5x10^{-32}$	<0.001	0.985	0.382
	KLKB1	$5.4x10^{-8}$	<0.001	-0.488	0.092
	KNG1	$1.1x10^{-7}$	<0.001	-0.479	0.097
	NTproBNP	$1.2x10^{-5}$	0.002	-0.380	0.061
rs3733402 in the *KLKB1* locus	KLKB1	$4.4x10^{-12}$	<0.001	-0.506	0.152
	KNG1	$5.2x10^{-5}$	0.034	-0.309	0.049
	NTproBNP	$4.2x10^{-8}$	<0.001	-0.393	0.098
	uPAR	$4.4x10^{-8}$	<0.001	-0.401	0.097

β, effect size of association in standard deviation units. R^2, amount of phenotypic variation explained by the variant.

American Heart Association. Circulation Cardiovascular Genetics

Figure Legends:

Figure 1: Overview of the three stages of association analyses. **A**) *cis*: for each of the 51 phenotypes (protein levels), we tested the variants located in the *cis* gene loci for associations with their respective protein level, **B**) *cis*-acting-in-*trans*: we tested the significant *cis*-pQTLs from stage 1 for *trans* effects against each of the 50 other protein levels, and **C**) *trans*: we tested all variants in the 50 *cis* loci (C3 and C3b share the same locus) for association with each of the 51 protein levels.

Figure 2: Association of *cis* variants with protein levels. Modified Manhattan plot showing the –log10 P-values for association between variants in each *cis* locus (interval encoding protein +/- 500kb) and the respective protein levels. The red dashed line indicates the study-wide significant P-value cutoff when only examining *cis* regions ($6.9x10^{-7}$) for a FWER <0.05.

American Heart Association. Circulation Cardiovascular Genetics

Figure 3: Schematic showing proteins with identified *trans* associations and their nominal associations with SNPs in *F12* and *KLKB1*. Previously known (solid) and proposed in this study (dashed) cleavage reactions are represented with arrows. Nominal P-values for the associations between protein levels and rs3733402 in the *KLKB1* locus and rs1801020 in the *F12* locus are shown respectively in orange and purple boxes next to the protein of interest.

Figure 4: Kallikrein cleaves proBNP *in vitro*. A) A silver stain of recombinant proBNP and kallikrein incubated together for 30, 60, and 90 minutes with and without a kallikrein-specific inhibitor (PPACK II) and B) a western blot of an identical experimental setup using an anti-BNP

antibody. The silver stain binds all protein present and is a more sensitive procedure than using the anti-BNP antibody for the western blot. We believe that this explains why the amount of proBNP in the +/+/- wells visually appears to be different between the silver stain and western blot.

American Heart Association

Circulation

Cardiovascular Genetics

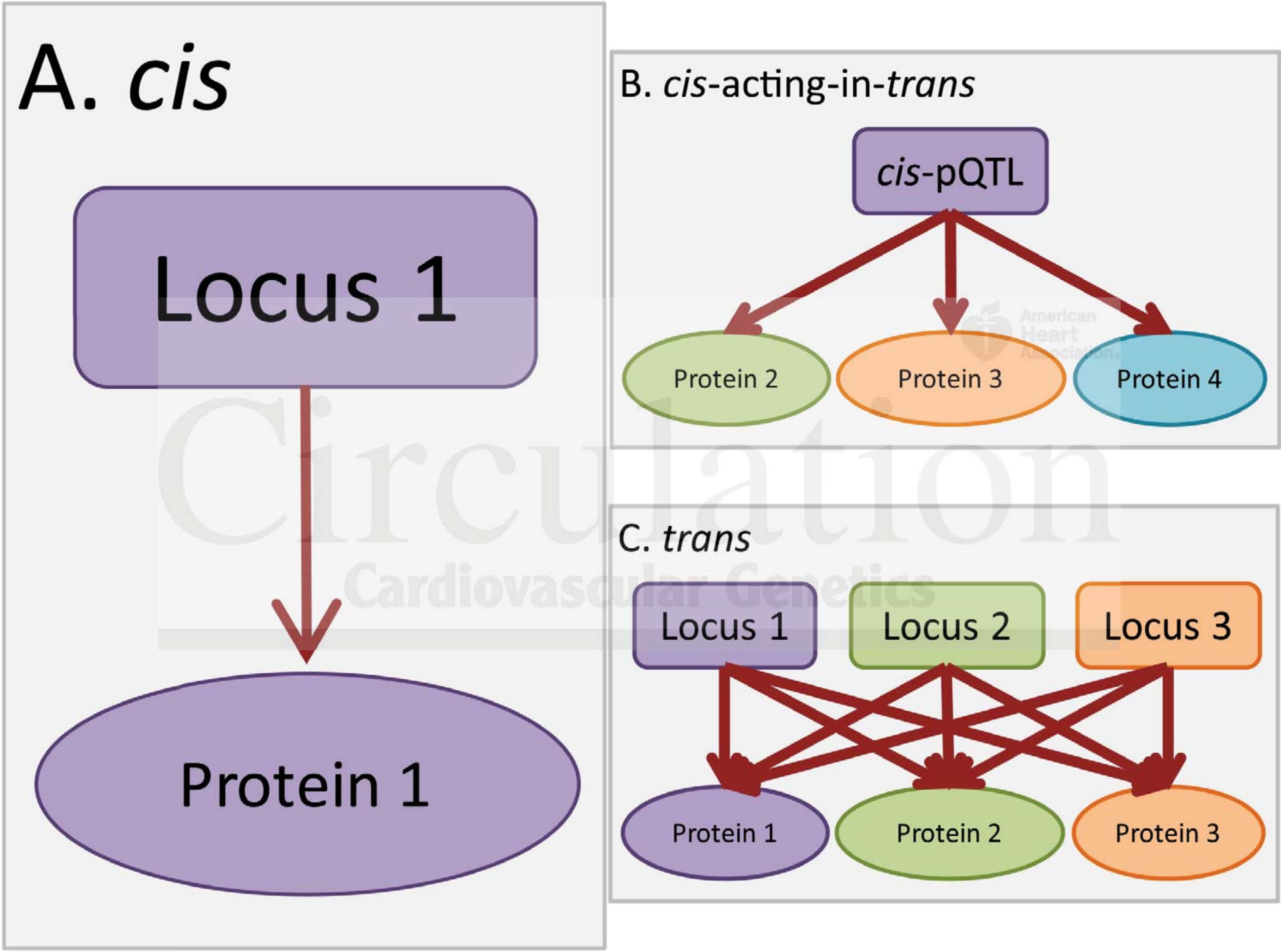

Circulation Cardiovascular Genetics
American Heart Association

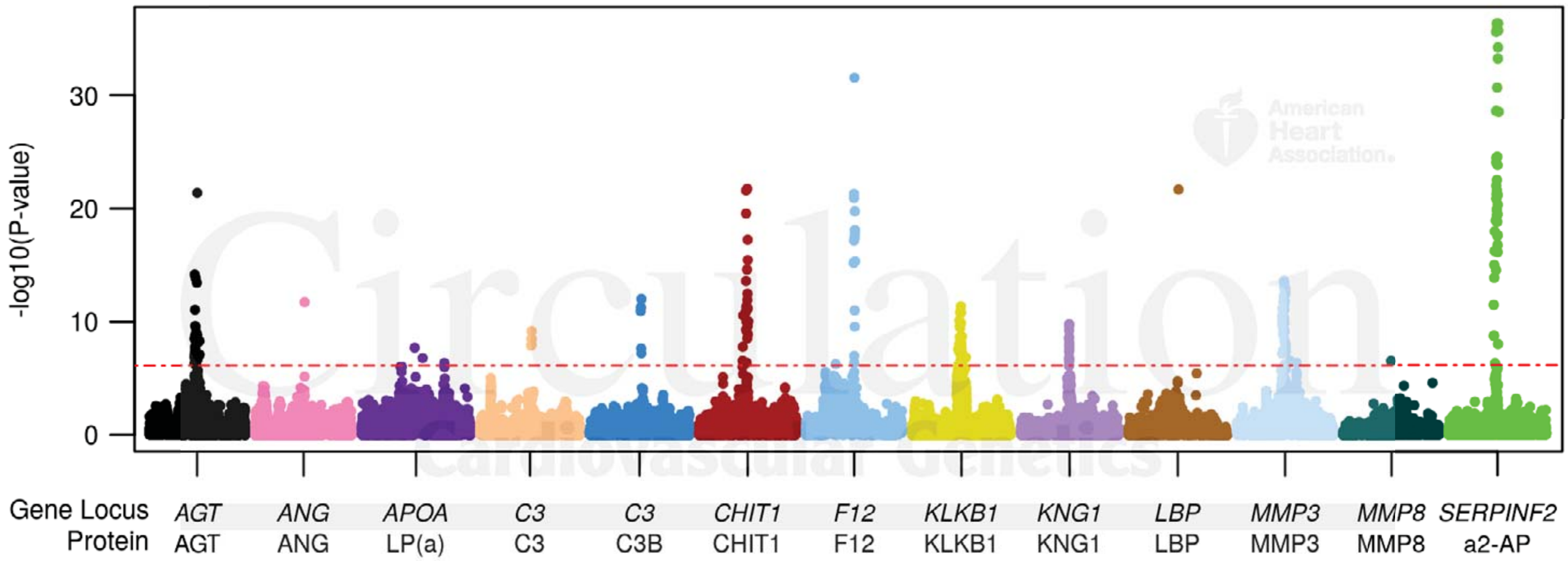

-log10(P-value)
30
20
10
0
Gene Locus
Protein
AGT
AGT
ANG
ANG
APOA
LP(a)
C3
C3
C3
C3B
CHIT1
CHIT1
F12
F12
KLKB1
KLKB1
KNG1
KNG1
LBP
LBP
MMP3
MMP3
MMP8
MMP8
SERPINF2
a2-AP

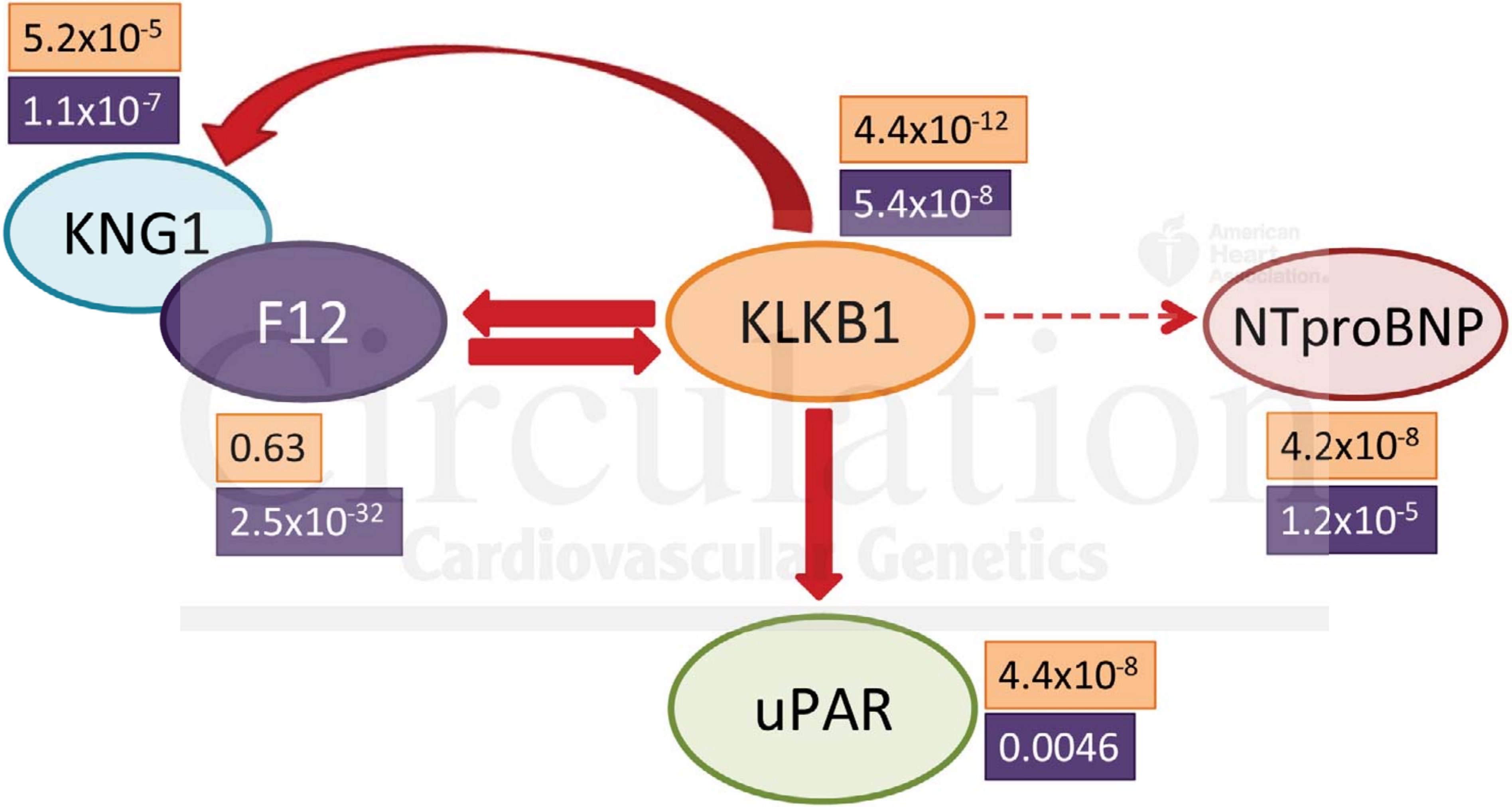

Circulation Cardiovascular Genetics
American Heart Association

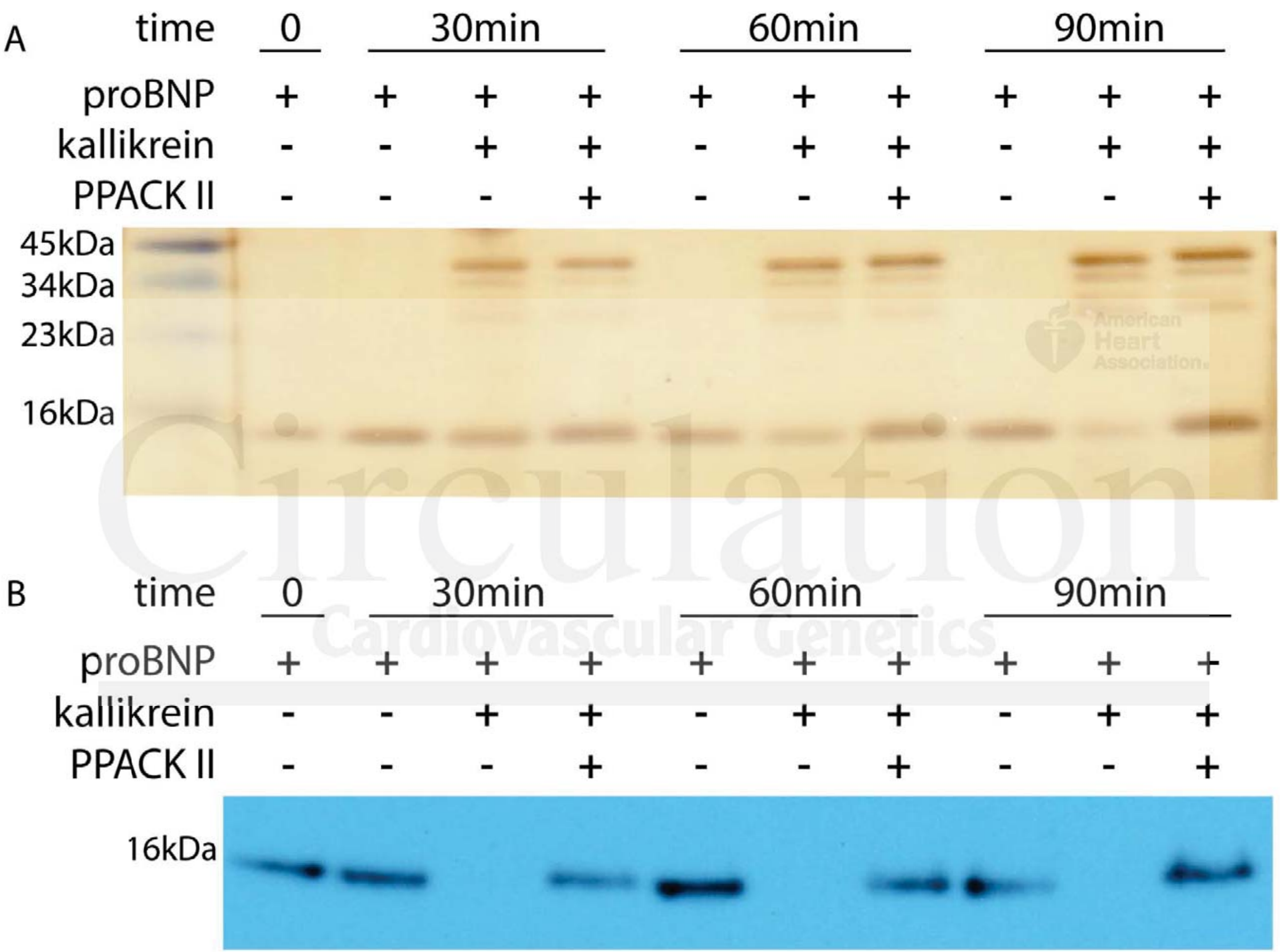

Circulation Cardiovascular Genetics
American Heart Association
